# A genome-scale *Escherichia coli* kinetic metabolic model k-ecoli457 satisfying flux data for multiple mutant strains

**DOI:** 10.1038/ncomms13806

**Published:** 2016-12-20

**Authors:** Ali Khodayari, Costas D. Maranas

**Affiliations:** 1 Department of Chemical Engineering, The Pennsylvania State University, University Park, Pennsylvania 16802, USA

## Abstract

Kinetic models of metabolism at a genome scale that faithfully recapitulate the effect of multiple genetic interventions would be transformative in our ability to reliably design novel overproducing microbial strains. Here, we introduce k*-*ecoli457, a genome-scale kinetic model of *Escherichia coli* metabolism that satisfies fluxomic data for wild-type and 25 mutant strains under different substrates and growth conditions. The k*-*ecoli457 model contains 457 model reactions, 337 metabolites and 295 substrate-level regulatory interactions. Parameterization is carried out using a genetic algorithm by simultaneously imposing all available fluxomic data (about 30 measured fluxes per mutant). The Pearson correlation coefficient between experimental data and predicted product yields for 320 engineered strains spanning 24 product metabolites is 0.84. This is substantially higher than that using flux balance analysis, minimization of metabolic adjustment or maximization of product yield exhibiting systematic errors with correlation coefficients of, respectively, 0.18, 0.37 and 0.47 (k-ecoli457 is available for download at http://www.maranasgroup.com).

## Abstract (web summary)

Kinetic models of microbial metabolism have great potential to aid metabolic engineering efforts, but the challenge of parameterization has so far limited them to core metabolism. Here, the authors introduce a genome-scale metabolic model of *E. coli* metabolism that satisfies fluxomic data for a wild-type and 25 mutant strains in various growth conditions.

## Graphical abstract

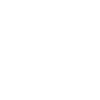

Rapid and pervasive advances in genome editing^1^ and copying^2^ techniques have dramatically reduced the time and cost^3^ for microbial strain construction. This has enabled probing multiple and often complex intervention strategies in pursuit of an overproducing cellular phenotype. Existing computational strain design tools operating at a genome scale^4^,^5^,^6^ primarily rely on a stoichiometric description of metabolism. Despite many successes^7^,^8^,^9^, designed mutants often fail^10^,^11^ as stoichiometric models do not directly account for enzyme level, metabolite concentration and substrate-level regulatory barriers. Kinetic models provide a computational vehicle for capturing these effects; however, they have been mostly descriptive in nature, parameterized for a single metabolic phenotype^12^ and are thus incapable of predicting the often systemic effect of metabolic perturbations. A number of efforts have been proposed that standardize kinetic expressions through approximation and provide a tractable parameterization workflow even at a genome scale (for example, a log-lin approach for *Escherichia coli*^13^ and a lin-log approach for yeast^14^). However, by design, these approaches are aimed at providing accurate production in the vicinity of the reference state. Kinetic models that satisfy multiple flux data sets^15^ have so far been limited in their metabolic scope capturing mostly central metabolism^15^,^16^.

Recent approaches first deconstruct complex kinetic expressions into their elementary steps^17^ and then reassemble them into kinetic expressions that capture all known substrate-level regulations. Uncertainty in assigning unique kinetic parameter values is circumvented by creating not only a single kinetic model, but rather an ensemble of kinetic models through parameter sampling^13^. The ensemble modelling (EM) approach^18^, for example, relies on the sampling of reaction reversibilities and enzyme fractions^19^ to create an ensemble of models. The original ensemble is subsequently ‘whittled down' by successively rejecting model parameterizations inconsistent with concentration or flux data for knockout mutants^18^. This approach ultimately leads to an empty ensemble if a large number of flux data for knockout mutants are imposed as required model output. A more efficient way to sample promising kinetic parameter space is achieved through integration of the EM procedure with a machine-learning inspired genetic algorithm (GA) that exchange the best reaction parameterizations across all models in the ensemble through the recombination operation^15^. This procedure was used to develop a core kinetic model (core model) of *E. coli* metabolism by seamlessly integrating flux data for a wild-type and seven mutant strains under aerobic conditions with glucose as the carbon substrate. The model was accurate in recapitulating genetic perturbations as long as growth conditions remained aerobic and the list of deleted genes was in the neighbourhood of the ones used during model parameterization^15^. In fact, a follow-up study revealed that the core model did worse than flux balance analysis (FBA) in predicting fluxes for fermentative (that is, anaerobic) growth^11^. These observations reaffirmed that, unlike FBA, kinetic model predictive ability is determined by the completeness of the modelled regulatory interactions and scope of imposed metabolic flux data in response to gene knockouts. Consequently, by increasing the set of flux data for mutants distributed across a wide range of pathways and augmenting the set of regulatory interactions, prediction fidelity is successively improved^11^,^15^ while subsuming the benefits afforded by a genome-scale level description. These benefits include a detailed description of biomass and global inventory of all metabolites and cofactors. These observations motivated this study, where both the scope of flux data sets and the complexity of regulatory interactions are significantly increased over previous efforts in an attempt to endow the kinetic model with sufficient detail to enable the reliable prediction of perturbed metabolic phenotypes.

The k-ecoli457 genome-scale kinetic model of *E. coli* metabolism was parameterized by combining a machine-learning algorithm^15^ and the EM formalism^18^. Model parameterization is performed by minimizing discrepancies between model predictions and experimentally measured steady-state flux distributions for 25 mutant strains including 21 genetically perturbed strains with glucose as the carbon substrate (19 under aerobic^20^ and two under anaerobic conditions^21^), and four with different carbon substrates under aerobic conditions (three with pyruvate^22^ and one with acetate^23^). Model predictions were tested against multiple experimentally measured data sets that were not used during model parameterization. These included (i) 898 steady-state metabolite concentrations for 20 of the mutant strains^20^,^21^,^22^,^23^, (ii) 234 Michaelis–Menten constants (185 *K*_*m*_ and 49 *k*_cat_ values) extracted from BRENDA^24^ and EcoCyc^25^ and (iii) 320 literature reported product yields for designed strains covering 24 different bioproducts. Comparisons revealed that 66% of the predicted metabolite concentrations as well as 51 and 63% of the estimated *K*_*m*_ and *k*_cat_ values, respectively, are within the experimentally reported ranges. This level of agreement of k-ecoli457 with experimental data exceeded the metrics reached by the core model^15^, despite the significantly increased scope of the model and coverage of fewer studied pathways. A primary reason for this prediction fidelity is that by directly imposing the biomass measurements in the model, the flux of over 30 reactions (coupled to biomass) in fatty acid and amino acid synthesis pathways is resolved. Notably, the average relative error of k-ecoli457 predictions for the product yield in 129 out of 320 designed strains is within 20% of the measured values. Stoichiometric model based techniques such as FBA, minimization of metabolic adjustment (MOMA) or maximization of product yield were within 20% of the experimentally reported yield for only 16, 18 and 65 of the designed strains, respectively. Overall, the predicted product yields by k-ecoli457 achieve significantly higher value of correlation with experimental data (that is, Pearson's correlation coefficient of 0.84) than FBA, MOMA or maximization of product yield (that is, 0.18, 0.37 and 0.47, respectively). These results quantitatively demonstrate that k-ecoli457 can reliably be used to predict genetically perturbed *E. coli* phenotypes under different growth conditions with a substantially higher accuracy than any other earlier modelling effort.

## Results

### k-ecoli457 construction and predictions

**Figure 1:**
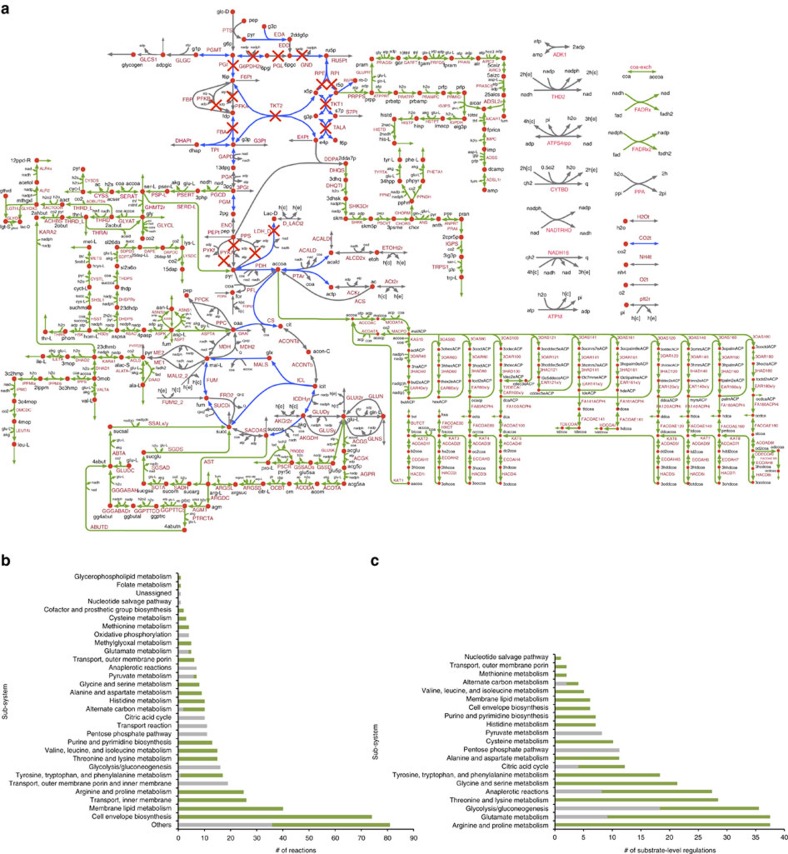
A representation of k-ecoli457 and the core model. (**a**) A pictorial representation of the k-ecoli457 model of *E. coli* metabolism. Red X's denote the location of reaction deletions in the mutant data sets. Reactions in the previously developed core model^15^ are shown in grey (no flux data) and blue (with flux data) while the additional reactions in k-ecoli457 are shown in green (no flux data). (**b**) Sub-system classification of reactions in the k-ecoli457 model. (**c**) Sub-system classification of the integrated regulatory interactions. Grey bars denote the content of the core model^15^, while green bars denote the additional reactions/regulations included in k-ecoli457.

The k-ecoli457 model contains 457 reactions and 337 metabolites and includes all reactions from the largest previously published mechanistic kinetic model of *E. coli*^15^ encompassing representations for most pathways from the genome-scale *i*AF1260 model (see Supplementary Data 1 for details). In general, out of 2,390 reactions in the *i*AF1260 model of *E. coli*, 1,603 reactions do not carry any flux, as they are either inactive (that is, 720 reactions) upon imposing the flux data of the reference (wild-type) strain or blocked (that is, 883 reactions)^26^. From the reactions that can carry flux, k-ecoli457 does not cover lipopolysaccharide and murein biosynthesis pathways as the molecular composition of some of these polymers is unknown and they only contribute <6% of the overall biomass^27^. In addition, 295 substrate-level regulatory interactions mined from enzyme biochemistry repositories such as BRENDA^24^ and EcoCyc^25^ were integrated in the model (see Fig. 1). In summary, k-ecoli457 is more than three times larger than that of the previously developed largest core kinetic model (core model)^15^ (that is, 138 reactions, 93 metabolites and 60 substrate-level regulatory interactions).

**Figure 2:**
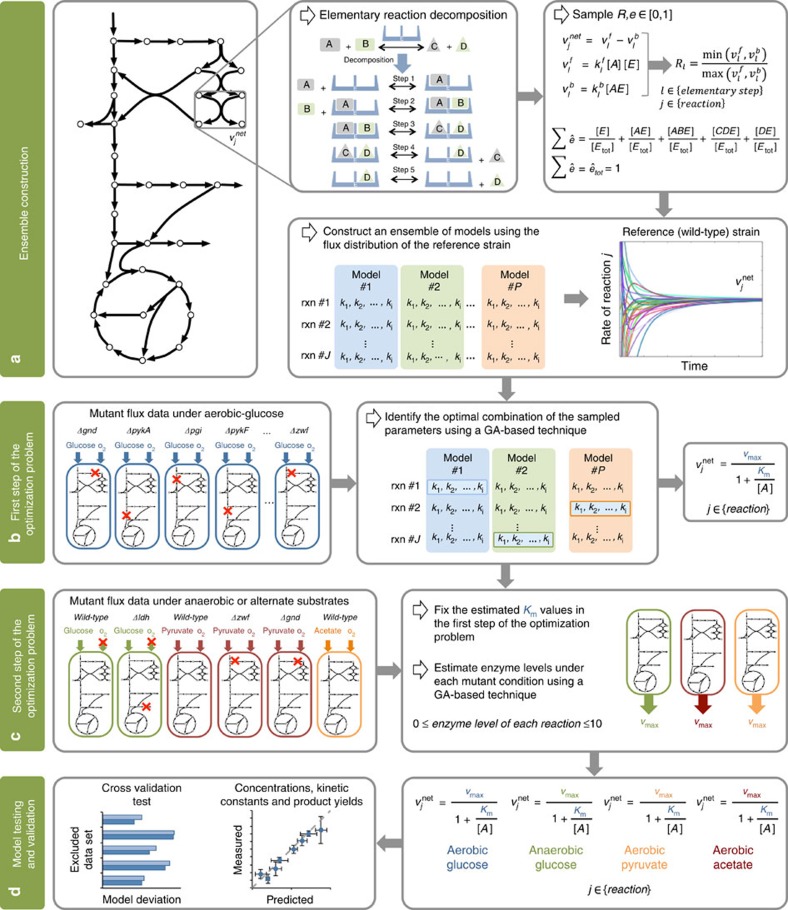
A schematic representation of k-ecoli457 construction procedure. (**a**) Reactions are decomposed into their elementary steps and an ensemble of *P* models is constructed by sampling enzyme fractions and reaction reversibilities. (**b**) The first step of the optimization problem identifies the optimal combination of the sampled parameters by minimizing k-ecoli457 prediction deviation from the measured flux data in the nineteen mutant strains grown aerobically with glucose. (**c**) The second step of the optimization problem estimates the level of enzymes under the other three mutant conditions by minimizing k-ecoli457 prediction deviation from the measured flux data in the remaining six mutant strains grown anaerobically with glucose, aerobically with pyruvate and aerobically with acetate. (**d**) Model predictions are tested and validated using cross-validation analysis and experimentally measured metabolite concentrations, Michaelis–Menten constants and product yields.

For the k-ecoli457 model parameterization, an initial ensemble of elementary kinetic models that converge to the steady-state flux distribution of the reference (wild-type) strain was constructed (see Fig. 2a). A two-step optimization procedure was used to parameterize k-ecoli457. The first step identified the equivalent Michaelis–Menten constants (that is, *K*_*m*_ and *v*_max_) using the experimentally measured flux data for the nineteen mutant strains grown aerobically with glucose. This is achieved by identifying the best combination of the sampled kinetic parameters in the ensemble (see Fig. 2b and Supplementary Methods, GA implementation). Next, the estimated parameters (that is, *K*_*m*_) were fixed and the levels of enzymes (that is, *v*_max_) were estimated under the other three growth conditions (that is, anaerobically with glucose, aerobically with pyruvate and aerobically with acetate), separately, by solving the second step of the optimization procedure (see Fig. 2c). In general, the parameterized k-ecoli457 model performs well by attaining the predicted fluxes within the experimental ranges for 61% of the reactions with measured flux data in the mutant strains (see Supplementary Data 2 for the predicted and measured flux data sets). In particular, we observed that k-ecoli457 predictions are often (that is, 73%) completely within the experimental error ranges for the reactions eliminated in the mutant strains, including those in the glycolysis and pentose phosphate (PP) pathways (that is, blue coloured reactions crossed in Fig. 1). This is because the metabolic response upon elimination of the reaction quantifies the extent of the reaction's effect on metabolism and assesses the plasticity of the reaction loss in other mutation scenarios. For the remaining reactions, k-ecoli457 predictions are between 2 and 3 s.d. of the reported experimental ranges for 79 and 88% of the reactions, respectively (see Supplementary Fig. 2).

We also performed leave-one-out and leave-two-out cross-validation analyses to assess the robustness of the estimated parameters in k-ecoli457 compared with that of the core model (see Supplementary Methods, cross-validation analysis). A comparison showed that core model predictions exhibited higher deviations (that is, by about threefold) compared with those of k-ecoli457 in predicting metabolic fluxes of the cross-validated mutant (that is, predictions within 5% of the original model in k-ecoli457 versus 15% in the core model). This implies that k-ecoli457 parameterization is significantly more robust compared with that of the core model. In general, the analyses revealed that a diverse set of mutant experimental data for different pathways under different growth conditions is required to achieve a robust model parameterization^15^. For example, we observed that the failure of model cross-validation for mutant Δ*pgi* by the previously published core model^15^ was resolved in k-ecoli457 through the presence of three additional mutant flux data sets (that is, Δ*pfkA*, Δ*pfkB* and Δ*fbaB*) that filled in information lost by removing the Δ*pgi* data set. Unsurprisingly, model parameterization was not as robust for the two mutant strains under anaerobic conditions (that is, wild-type and Δ*ldh*) as alluded by higher prediction deviations from experimental data upon removal of one of the two data sets (that is, a 14% increase in average scaled relative deviation). Because fluxes of the fermentative products (that is, formate, lactate, acetate and ethanol) are significantly different between the two data sets, both data sets are needed as they cannot complement information upon the loss of one of the two. These discrepancies propagate to some extent in other parts of the network, however, model prediction is still adequate for the rest of reactions (that is, an average 8% deviation from the experimental ranges).

### Comparison of the estimated elementary kinetic parameters

We first compared the estimated values for each individual elementary kinetic parameter in k-ecoli457 with those in the previously published core model^15^ by defining a confidence range of 10% from the estimated parameter values (see Supplementary Data 2 for the estimated parameters). The comparison revealed significant differences, as for 90% of the elementary kinetic parameters there was no overlap between the estimated ranges. The majority (that is, 63%) of the elementary kinetic parameters that changed in value significantly belong to reactions utilizing cofactors (that is, atp, adp, amp, nad(h), nadp(h)). This is due to the fact that the core model^15^ could not accurately track the concentration of cofactors as only a limited number of reactions that contribute to the cofactor balance were included. For example, amino acid and membrane lipid metabolism reactions were absent in the core model^15^. Thus, errors in the estimation of cofactor concentrations propagated to the corresponding elementary kinetic parameter estimates.

### Comparison of predicted Michaelis–Menten parameters

**Figure 3:**
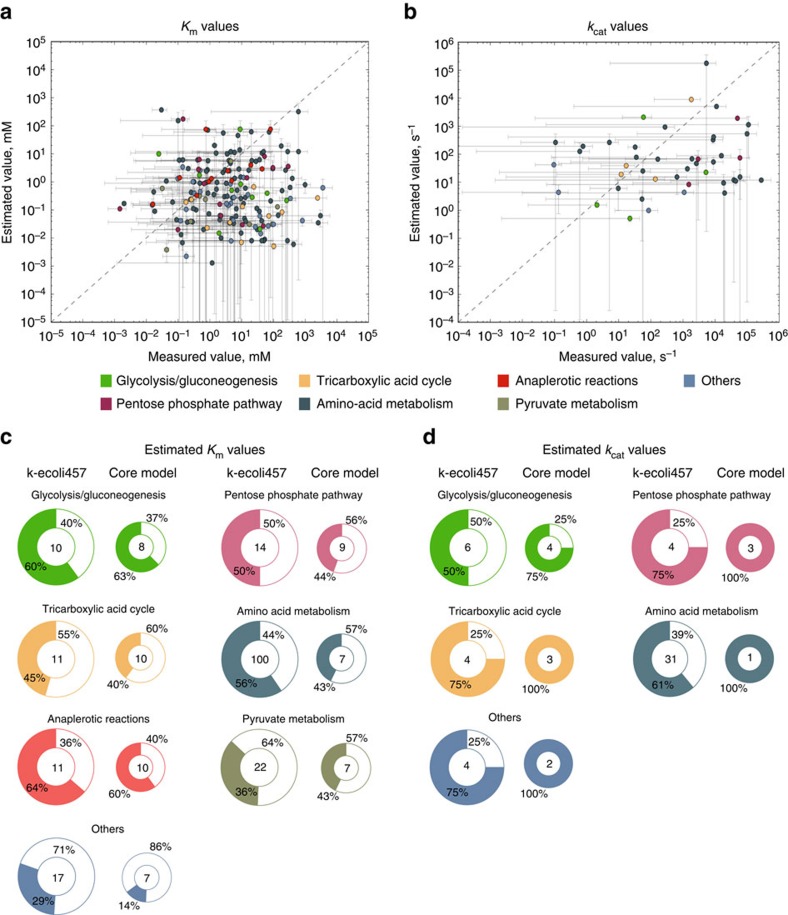
Estimated and measured kinetic parameters. Comparison of the computed (**a**) Michaelis constant *K*_*m*_ and (**b**) turnover number *k*_*cat*_ in k-ecoli457 and the values reported in BRENDA^24^ and EcoCyc^25^. The error bars denote 1 s.d. confidence interval for the corresponding parameter, the dots represent the mean value of the measured versus computed parameters and dashed lines show equal predicted and measured parameters. Comparison of the computed (**c**) *K*_*m*_ and (**d**) *k*_cat_ in k-ecoli457 (left) and the core model (right)^15^. The numbers within the circle plots indicate the number of measured parameters and the darker sectors represent the portion of the parameters whose estimated ranges overlap experimentally reported ranges.

To assess the accuracy of the estimated elementary parameters, we compared the corresponding Michaelis–Menten constants with experimental values from BRENDA^24^ and EcoCyc^25^. In accordance with the EM approach, the elementary kinetic parameters and therefore the Michaelis–Menten constants, are scaled by the corresponding metabolite concentrations in the reference (that is, wild-type) strain. We used the experimentally reported concentration data for the wild-type strain^20^,^28^ to rescale the estimated Michaelis–Menten constants. We extracted 234 measured Michaelis–Menten constants including 185 *K*_*m*_ and 49 *k*_cat_ values for the reactions present in k-ecoli457 (see Supplementary Data 2 for the estimated and measured ranges). In general, the results showed that 51% of the estimated *K*_*m*_ values and 63% of the *k*_cat_ values in k-ecoli457 overlap with the reported experimental value ranges (see Fig. 3a,b). For the parameters shared by both the core model and k-ecoli457, the majority (that is, 77% for *K*_*m*_ and 86% for *k*_cat_) of the predictions within the confidence ranges in the core model were predicted again within ranges by k-ecoli457. Notably, the computed Michaelis–Menten constants in k-ecoli457 for amino acid and pyruvate metabolism exhibited significantly higher agreement with experimental data compared with the values in the core model^15^ (see Fig. 3c,d). In addition, we performed the same comparisons for only those parameters whose confidence ranges did not exceed two orders of magnitude which included 120 *K*_*m*_ and 14 *k*_cat_ parameters. Comparisons revealed that for this set 36% of the estimated *K*_*m*_ and 29% of the estimated *k*_cat_ values are within the experimentally reported ranges. Overall, these analyses demonstrate the importance of integrating pathways distant to central metabolism into the model as they can affect the quality of parameterization through the pools of shared metabolites such as cofactors. This motivates the development of flux data sets in response to genetic perturbations distal from central metabolism pathways to improve overall model parameterization. It is also important to note that the large magnitude in the confidence ranges for both measured and computed *k*_cat_ values detracts from the confidence for some of the estimated *k*_cat_ values.

### Comparison of predicted concentrations against unused data

**Figure 4:**
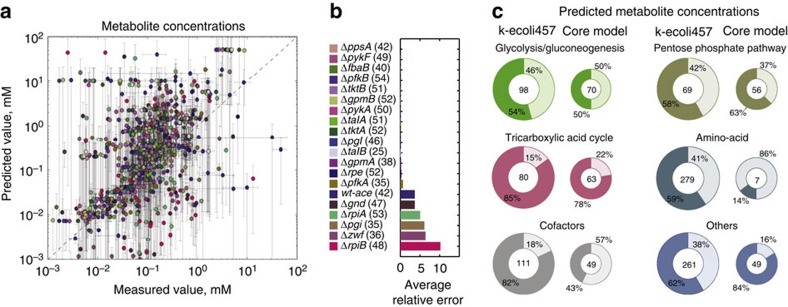
Predicted and measured metabolite concentrations. (**a**) Comparison of the predicted steady-state metabolite concentration in k-ecoli457 and the experimentally reported values^20^,^23^. The error bars denote 1 s.d. confidence interval for the corresponding concentration, the dots represent the mean value of the measured versus predicted parameters and dashed line shows equal predicted and measured concentrations. (**b**) The average relative error of the predicted metabolite concentrations in the twenty mutant strains. The number next to each mutant represents the number of metabolites with measured concentration. (**c**) Comparison of the predicted steady-state metabolite concentration in k-ecoli457 (left) and the predicted values in the core model (right)^15^. The numbers within the circle plots indicate the total number of metabolites with measured concentration in the twenty mutant strains and the darker sectors represent the portion of the metabolites whose predicted concentration ranges overlap experimentally reported ranges.

Similar to the Michaelis–Menten constants, the predicted normalized steady-state metabolite concentrations of the mutant strains were first recast as actual concentrations. In total, we extracted 898 experimentally measured concentrations spanning ∼45 metabolites in twenty mutant strains (a total of 294 concentrations in the core model^15^). The comparison showed that 66% of the predicted metabolite concentration ranges overlap with the experimentally reported ranges (see Fig. 4a and Supplementary Data 2 for the predicted and measured concentration data sets). We note that for the metabolite concentrations present in both the core and k-ecoli457 models, the majority of concentrations (that is, 80%) that were within the confidence ranges in the core model^15^ were again predicted within the ranges by k-ecoli457. In particular, we observed k-ecoli457 achieved higher accuracy for metabolites in pathways challenged with more mutant flux data (that is, pyruvate metabolism, the tricarboxylic acid (TCA) cycle and the PP pathway). For example, the predicted concentration ranges of the commonly measured metabolites acetate, lactate, succinate, malate, fumarate, ribose-5-phosphate and xylulose 5-phosphate by k-ecoli457 showed overlap with experimental ranges in the 20 mutant strains for 80% of the measured concentrations, while the core model predictions showed overlap for only 31% of the measured concentrations (see Fig. 4c). This highlights the efficacy of parameter estimation for reactions directly affected by the mutations in the training data sets. In addition, energy and cofactor regulatory information, whenever available in BRENDA or EcoCyc (that is, 64 regulations), were included to account for the substrate-level effect of redox and energy regulation in k-ecoli457. In general, we observed more accurate predictions for the concentration of cofactors (that is, nad(h), atp, amp and adp) with k-ecoli457 compared with the core model^15^ (82% versus 43% of the predicted concentrations overlap with experimental ranges, respectively). This is because a more complete integration of cofactor utilizing pathways in k-ecoli457 led to more accurate component balances, therefore, yielding more accurate concentration predictions. The accuracy of prediction, however, was often limited to the metabolites in central metabolism. For example, a high-prediction deviation was observed for metabolites in amino acid metabolism in Δ*pgi,* Δ*gnd,* Δ*zwf,* Δ*rpiA,* Δ*rpiB* and wild-type strain grown with acetate (see Fig. 4b). This is a manifestation of the lack of flux data sets for the associated pathways during model parameterization. In addition, the important transcriptional regulation of cellular growth on the oxidative section of the PP pathway^29^ was not captured in k-ecoli457. This led to erroneous predictions for the aromatic amino acid concentrations in five knockout mutants. Even with missing experimental data and regulatory interactions, we note that 59% of the predicted concentrations for the metabolites in amino acid metabolism are still well within the experimental measurement error ranges. In fact, this tight coupling of the metabolite concentration and kinetic parameters through reaction kinetics leads to accurate predictions by providing mutual backup for missing information. In addition, a comparison between the predicted and measured concentrations with confidence ranges no greater than two orders of magnitude (that is, 786 concentrations) revealed that 63% of the estimated concentrations are still within the experimentally reported ranges.

### Predicted product yield for 320 overproducing mutants

We extracted experimentally reported yields of overproduction for 320 different engineered *E. coli* strains for a diverse range of bioproducts from 47 separate studies and compared them with k-ecoli457, FBA, MOMA and maximization of product yield predictions (see Methods for calculation). The target products included 24 different chemicals under both aerobic (184 mutants) and anaerobic (136 mutants) conditions spanning biofuels (ethanol, butanol and isobutanol), pharmaceuticals and nutraceuticals (artemisinin and naringenin), polymer precursors (hydroxybutyrate, hydroxystyrene, butanediol, acetate, formate, glucaric acid and styrene) and commodity and specialty chemicals (lycopene, indigo, malate, fumarate, succinic, cinnamic and muconic acids) (see Methods and Supplementary Data 2).

**Figure 5:**
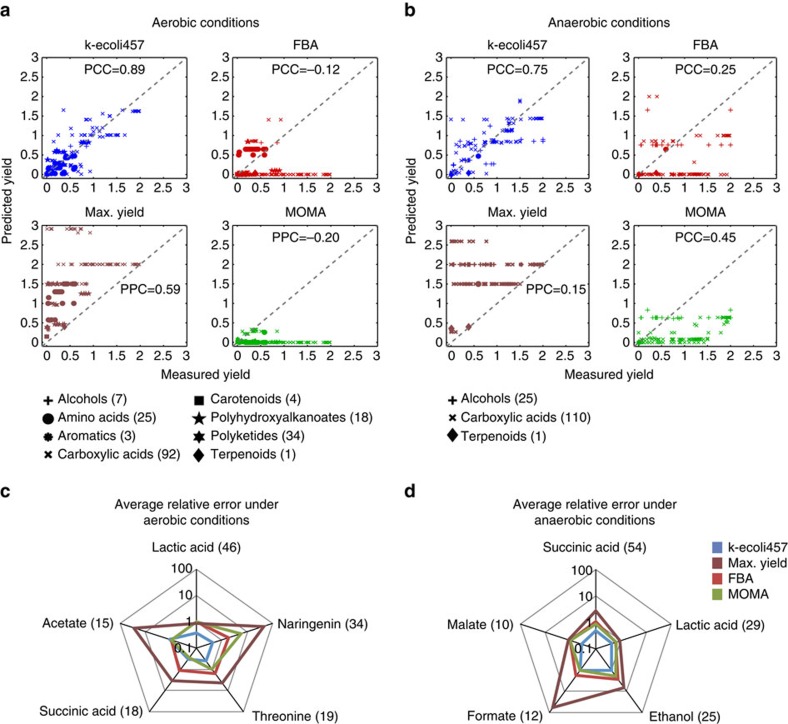
Predicted and measured product yields. Comparison of the experimentally reported yield of overproduction for 320 different engineered *E. coli* strains and k-ecoli457, FBA, MOMA and maximization of product yield predictions under (**a**) aerobic and (**b**) anaerobic conditions. PCC is the Pearson's correlation coefficient between the predicted product yield values and experimental data. Dashed lines show equal predicted and measured yields and the number next to each category represents the total number of designed strains extracted from literature. Average relative error is displayed between k-ecoli457 predictions and experimental data under (**c**) aerobic and (**d**) anaerobic conditions for the chemicals with at least ten data points. The number next to each chemical shows the number of designed strains extracted from literature. The reported average relative error was calculated as the average relative difference between the measured and predicted product yield values (product mol per glucose mol).

The Pearson's correlation coefficients between the experimental data and product yield predictions by k-ecoli457, FBA, MOMA and maximization of product yield, respectively, are 0.84, 0.18, 0.37 and 0.47 (*P*<10^−3^). A comparison also revealed that the product yield in 129 out of 320 designed strains are predicted within 20% of the experimental measurements by k-ecoli457, whereas FBA, MOMA and maximization of product yield predicted the product yield for only 16, 18 and 65 of the designed strains, respectively, with the same accuracy. In general, under aerobic conditions k-ecoli457 closely reproduces the yields for products that branch out from the PP pathway (for example, naringenin and muconic acid), the TCA cycle (for example, malate, succinate, fumarate, threonine and butanol) and pyruvate metabolism (for example, lactate, isobutanol and 2,3-butanediol). Both FBA and MOMA tend to underestimate their yields as more carbon is diverted towards biomass (see Fig. 5a) with the exception of naringenin and threonine where FBA overestimates their yield as it is blind to substrate-level regulatory interactions. For the naringenin engineered strain, FBA does not capture two strong feedback inhibitions of tyrosine and phenylalanine on the chorismate pathway^30^, thus pooling additional arabino-heptonate 7-phosphate (2dda7p) towards the flavanone pathway. Likewise, regulatory inhibitions in glycolysis, chorismate and the PP pathways limit flux towards erythrose 4-phosphate, phosphoenolpyruvate and shikimate. The same inability to capture multiple regulatory interactions in the aspartate, lysine, asparagine, methionine and isoleucine pathways that share precursor oxaloacetate with the threonine pathway leads to an over prediction for the threonine yield by FBA. Maximization of product yield not surprisingly typically overestimated experimental product yield. Product yields under anaerobic conditions were again tracked better by k-ecoli457 as both FBA and MOMA often underestimate them (65 and 82% of strains, respectively) by directing all carbon flux towards biomass (see Fig. 5b). We find that reliable product yield prediction by k-ecoli457 requires both the change in the enzyme level under anaerobic conditions and the activation of relevant substrate-level regulations. For example, for lactate, malate and succinate overproducing mutants, the experimentally reported high activities of the glyoxylate shunt^31^ and reductive section of the TCA cycle^32^ were correctly captured by the updated enzyme levels under anaerobic conditions (see Supplementary Data 2).

k-ecoli457 often underestimates the yield of acetate under aerobic conditions as well as ethanol and formate under anaerobic conditions (see Fig. 5c,d) due to inadequate training flux data sets involving mutants of the respective pathways. For example, only two mutant flux data sets under anaerobic conditions (that is, a wild-type and Δ*ldh*) were available for model parameterization implying insufficient training data as affirmed in the cross-validation analysis (see Supplementary Methods, cross-validation analysis). Another important factor that is often overlooked is the lack of flux data sets that span multiple growth phases or dilution rates, and not just early growth phase or low dilution rates. In particular, acetate production is poorly captured by k-ecoli457 as the fluxomic information for all nineteen mutant strains under aerobic glucose conditions were measured under low dilution rates (that is, 0.2 h^−1^), while product yield data were often reported in higher dilution rates. These observations identify the limits of prediction and pinpoint needed flux measurements under different dilution rates, growth phases, alternate substrates and anaerobic conditions using both batch^33^ and continuous cultures^16^. Nonetheless, despite of data scarcity, k-ecoli457 predictions remain substantially better than FBA, MOMA and maximization of product yield. In general, we observed that both FBA and MOMA predictions demonstrated reasonable agreement with experimental data only whenever the targeted product is energy/redox coupled with biomass and measured product yield value scaled by the maximum theoretical yield was relatively low (that is, <0.2). The maximization of product yield better reflected the measured product yields whenever the scaled product yield value was relatively high (that is, higher than 0.9). Whenever the designed mutant's product yield did not significantly differ from wild-type, MOMA performed better than FBA (for example, lycopene and styrene under aerobic conditions).

## Discussion

Here, we developed k-ecoli457, a kinetic model of *E. coli* metabolism that approaches genome-scale coverage (457 reactions and 337 metabolites). Comparisons of k-ecoli457 with a previously constructed core model^15^ revealed significant improvement in prediction accuracy despite the significantly expanded model scope and the corresponding paucity of fluxomic data for distal pathways. We found that the global inventory of highly participating metabolites (that is, cofactors), the large number of resolved reaction fluxes coupled to the biomass measurement and the complete description of the proportions of metabolites sequestered within biomass contributed to the prediction improvements in k-ecoli457. A comparison between predicted fluxes, however, revealed that the average relative error of k-ecoli457 when applied to only the core reactions is higher compared with the core model (3 versus 10%). This is because the core model has been tuned exclusively for these reactions whereas k-ecoli457 must describe four times more fluxes in (25 versus 7) experimental data sets. Cofactor concentrations in k-ecoli457 now participate in hundreds of reactions making it very difficult to pinpoint a unique value that matches all experimental data. Significant uncertainty in the experimental data sets across multiple pathways also contributes to the inability to perfectly match the core reaction set. Despite these challenges, 61% of the predicted fluxes by k-ecoli457 (78% in the core model) are within 1 s.d. of the experimental data. In addition, the agreement of the experimental yields (see Fig. 5) provides additional confidence for the robustness of the developed model. Prediction deficiencies remained for pathways lacking metabolic flux data sets in response to genetic perturbations (for example, membrane lipid metabolism and ED pathways). For example, we observed that even after the inclusion of additional flux data sets for k-ecoli457 parameterization compared with that of core model, the activity of the ED pathway was not properly captured. As the majority of the training flux data sets (that is, 22 out of 25) had an inactive ED pathway, the k-ecoli457 model predicted the same. While simple inclusion of additional data sets with nonzero ED flux may have rectified this limitation, this *a posteriori* correction may not be a fair representation of the proposed model and methodology. These limitations are likely to be ameliorated as expanded metabolomic (for example, MetaboLights^34^) and fluxomic (for example, CeCaFDB^35^) data sets are becoming increasingly available. Given data sets that span the metabolic capabilities of *E. coli*, the proposed machine-learning inspired parameterization strategy demonstrated that it is indeed possible to train a single model to predict the genetic and environmentally perturbed phenotypes with fidelity. In the same spirit, the same multi-data set parameterization concepts can be leveraged for applications of kinetic models in personalized healthcare^36^, biomarker identification^37^, drug discovery^38^ and modelling of microbial communities^39^.

Remaining challenges not addressed in this effort include allowing for substrate(s) uptake rates to become an output of the kinetic model. In k-ecoli457 all mutant fluxes in the training data sets were scaled with the corresponding substrate uptake rate. Given substrate uptake rates data sets^24^,^40^ for different mutations and growth conditions, a kinetic formalism that describes carbon uptake could be constructed and parameterized largely independent of internal reactions. In addition, large-scale metabolomic data sets for absolute or even relative concentrations^34^ can directly be ported in the machine-learning algorithm to further constrain model parameter values. This was not attempted here as we chose to treat metabolomic data *a posteriori* as a model consistency check. In addition, the assembled compilation of experimental product yields for 320 designed strains could serve as a starting point for more comprehensive compilations^41^ that will help to fairly assess follow-up efforts aimed at improving the accuracy and coverage of k-ecoli457. Kinetic model parameterization using such comprehensive data sets, however, must be carefully interpreted. For example, there exists substantial evidence for the presence of pathway channelling^42^,^43^,^44^ in metabolism as a mechanism for increasing the local concentration of metabolites and thus boost reaction rates (for example, channelling of glycolysis intermediates in *E. coli*^45^). As a result, if the relevant metabolite participates in other reactions, then the kinetic model will simply pool all the ‘local' concentrations of the metabolite within a single ‘average' concentration. This difference between local and average concentrations will propagate in the value of *k*_cat_ so as the reaction flux value is matched. This means that the values of the estimated metabolite concentrations and *k*_cat_ values may not always reflect *in vivo* kinetics but rather represent cell-averaged values. In addition, many other factors such as growth stage of the strain or even experimental group carrying out the fluxomic analysis can affect the quality and reproducibility of the flux data sets. These factors can lead to different flux data sets for exactly the same genotype (for example, different flux distributions for wild-type strain in ref. 20 versus ref. 46 or the different effect of *pfkA* and *pfkB* knockouts on glycolytic activity in ref. 20 versus ref. 47). Including conflicting flux data sets would cause significant problems in parameter estimation as the model will try to match the average values between the two data sets that are likely not physiologically relevant. Accounting for such variations remains an important topic to be addressed in follow-up studies.

Looking beyond substrate-level regulation, the increasing availability of data sets that provide genome-wide collections of interactions between mRNAs^48^, proteins^49^ and metabolites^50^ has dramatically expanded our knowledge of transcriptional, (post)translational and substrate-level interactions. Although the relative contribution of each of these regulatory layers is likely to be context dependent^51^, systematic implementation of regulatory events across multiple layers will ultimately be needed. Successful implementation of allosteric modification^52^ and substrate-level regulation^11^ with elementary kinetic mechanisms described in this paper establish a foundation for including additional layers. Developing efficient methods for reducing the complexity of models into more manageable ones without any information loss would also increase usability and community acceptance. This will also reduce computation complexity of integrating kinetic information into computational strain design protocols^4^,^11^. In particular, we have recently integrated 36 reactions with a kinetic description in the core model for strain design using the k-OptForce procedure^11^. Moving towards strain design with a full kinetic representation will ultimately require advances in solution techniques and accelerated ways of reaching steady-state fluxes and concentrations.

## Methods

### Model scope and initial ensemble construction

A metabolic model of *E. coli* metabolism composed of 457 reactions and 337 metabolites was constructed (see Fig. 1a and Supplementary Data 1). This model accounts for all reactions from the genome-scale *i*AF1260 model^27^ that carry flux under the experimental conditions of the flux measurements except lipopolysaccharide and murein biosynthesis. These include reactions in glycolysis/gluconeogenesis, the PP pathway, the TCA cycle, anaplerotic reactions, amino acid synthesis/degradation, fatty acid oxidation/synthesis and a number of reactions in other parts of the metabolism, such as co-factor and alternative carbon metabolism, membrane lipid and cell envelope synthesis and oxidative phosphorylation pathways (see Fig. 1b). In addition, 295 regulatory interactions were extracted from BRENDA^24^ and EcoCyc^25^ and included in the model by introducing new regulatory reactions (see Fig. 1c) (see Supplementary Data 1 for a comprehensive list)^53^. A simplified version of the biomass equation for *E. coli* described in (ref. 54) was also integrated into the model including all amino acids as well as other biomass constituent precursors in the central metabolism. This biomass equation was only used to ‘drain' biomass precursors from the pathways absent in k-ecoli457 (see Fig. 1).

Following the EM procedure^19^, all metabolic reactions and regulatory interactions in the model were first decomposed into their elementary steps and a mass action kinetic was developed for each elementary reaction. Elementary kinetic parameters were next expressed in terms of elementary reaction parameters, reaction reversibilities *R* and enzyme fractions 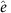, both bounded between zero and one. Reaction reversibility is defined as the elementary rate of the backward reaction divided by the forward reaction for each elementary step (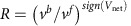 where *V*_net_ is the net flux of the reaction). Enzyme fraction is defined as the level of unbound enzyme *e* normalized by the total pool of the specific enzyme (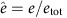 and 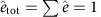). Metabolite concentrations were also normalized using the reference (wild-type) strain values. Elementary kinetic representations and scaled metabolite concentrations provide a convenient scaling between zero and one for parameter sampling for enzyme fractions and reaction reversibilities consistent with thermodynamic principles^19^. We used experimentally measured flux data for a wild-type *E. coli* strain growing aerobically with glucose^20^ as the reference strain for the ensemble scaling and construction.

The reference flux distribution was obtained by first maximizing biomass yield (per 100 mmol g DW^−1^ h^−1^ of glucose uptake and 200 mmol g DW^−1^ h^−1^ of oxygen uptake) in the *i*AF1260 model using FBA while imposing the experimental flux measurements (for 43 reactions)^20^ as constraints. Next, flux variability analysis^55^ was used to identify the flux ranges for reactions without experimental measurements upon fixing the biomass flux at the value obtained in the first step. The obtained flux ranges were then used to constrain the reactions without available measurements. A feasible flux distribution was then obtained by imposing the experimental data along with flux variability analysis-driven flux ranges in our model by maximizing biomass yield. This flux distribution was then used to anchor steady-state fluxes during the uniform sampling of the model parameters and elementary kinetic ensemble construction. We used an ensemble size of 2^17^=131,072 models, as no improvement in model predictions and convergence to the optimal solution was achieved for a larger ensemble size.

### Multiple flux data sets for model parameterization

Model parameterization was carried out using steady-state flux data for 25 mutant strains of *E. coli* under aerobic as well as anaerobic conditions with multiple carbon substrates. In particular, the flux data sets include nineteen single knockout mutant strains growing under aerobic conditions (that is, Δ*pgi,* Δ*pykA,* Δ*pykF,* Δ*ppsA,* Δ*gnd,* Δ*zwf,* Δ*rpe,* Δ*pfkA, *Δ*pfkB,* Δ*fbaB,* Δ*gpmA,* Δ*gpmB,* Δ*pgl,* Δ*rpiA,* Δ*rpiB,* Δ*talA,* Δ*talB,* Δ*tktA* and Δ*tktB*)^20^ and two strains growing under anaerobic conditions (that is, a wild-type and Δ*ldh*) with glucose as the carbon substrate^21^, three strains growing under aerobic conditions with pyruvate as the carbon substrate (that is, wild-type, Δ*zwf* and Δ*gnd*)^22^ and one strain growing under aerobic conditions with acetate as the carbon substrate (that is, wild-type)^23^. In total, we extracted flux measurements for ∼30 reactions in each mutant strain including the major intracellular reactions in glycolysis, the PP pathway and the TCA cycle (see Fig. 1a)^20^. In addition, we performed a coupling analysis with the *E. coli* biomass reaction^56^ to augment the set of flux data beyond those in central metabolism. We found that between 33 and 36 reactions (depending on the specific mutant) were fully coupled with biomass production and were added to the list of measured fluxes. These reactions were, not surprisingly, located in biomass constituent biosynthesis pathways, such as cell envelope metabolism and amino acid synthesis pathways (that is, threonine, lysine, methionine, valine, leucine, isoleucine, tyrosine, tryptophan, phenylalanine and histidine metabolism) (see Supplementary Data 1).

### Confidence intervals of the estimated and measured parameters

Confidence intervals were provided only for the measured fluxes of the wild-type strain in the training data sets^20^. Consequently, we used the same confidence intervals reported for each reaction in the wild-type strain to construct a flux range (that is, 1 s.d. confidence interval) around the reported values in the mutant strains. Likewise, for the metabolites with no reported confidence range in the mutant strains, we used the same confidence intervals reported for each metabolite in the wild-type strain to construct a concentration range (that is, 1 s.d. confidence interval). In addition, for the experimentally measured Michaelis–Menten constants (that is, *K*_*m*_ and *k*_cat_) with multiple reported values in BRENDA^24^ and EcoCyc^25^, confidence intervals were calculated using 1 s.d. from the mean of the reported parameter values. For parameters with only a single measured value we used a range of 10% from the reported parameter values as confidence intervals (see Supplementary Data 2). In addition, the predicted normalized metabolite concentrations and Michaelis–Menten constants were scaled by the corresponding metabolite concentration confidence ranges in the reference (wild-type) strain to convert them into the actual ranges (see Supplementary Methods, conversion of estimated elementary kinetic parameters). To reduce the uncertainties of the base concentrations, we extracted seven additional measured concentration data sets for the wild-type strain in addition to the reference strain data and used their confidence range intersections as the base ranges^16^,^28^,^33^,^57^,^58^,^59^,^60^ (see Supplementary Table 2). For metabolites with no concentration data in the wild-type strain (that is, 12%) we used the reported ranges in the *i*AF1260 model of *E. coli*^27^. Next, the agreement quality is gauged as an overlap between the reported and predicted confidence ranges.

### Estimation of k-ecoli457 parameters

Model parameterization was performed in two stages: (a) estimation of the elementary kinetic parameters and isozyme activity using the experimentally measured flux data for the single knockout mutants growing under aerobic minimal glucose conditions (that is, a total of nineteen flux data sets); and (b) estimation of enzyme levels under minimal glucose fermentative (anaerobic) conditions and alternate carbon substrates pyruvate and acetate (that is, a total of six flux data sets).

### Estimation of elementary kinetic parameters and isozyme activity

A GA implementation was used that minimized the deviation of the model predictions from the available flux measurements for the nineteen mutant strains grown aerobically with glucose^15^. In essence, this optimization based approach identifies the optimal combination of the sampled parameters and consequently the equivalent *K*_*m*_ and *v*_max_ values in Michaelis–Menten description by permuting parameterizations for different reactions across models in the ensemble. The consequence of an enzyme knockout is captured by fixing the total pool of the normalized enzyme level 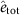 to zero (that is, 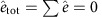) for the deleted reaction, while 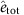 is assumed to remain unchanged for the remaining reactions. For the reactions catalysed by isozymes, deletion of one isozyme reduces (total enzyme) 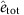 to a level between zero and the reference level (that is, 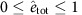), as it is not clear the extent at which the remaining isozyme can complement for the lost activity. Therefore, we allowed the minimization of the model predictions with experimental measurements to arrive at the best value for 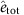 while maintaining the sum of 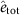 for all the isozymes to one. In total, we allowed the normalized enzyme levels 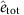
for seven reactions catalysed by 12 isozymes to vary (that is, Δ*pfkA, *Δ*pfkB,* Δ*fbaB,* Δ*gpmA,* Δ*gpmB,* Δ*pgl,* Δ*rpiA,* Δ*rpiB,* Δ*talA,* Δ*talB,* Δ*tktA* and Δ*tktB* under aerobic conditions with glucose as the carbon substrate) during step one (see Supplementary Methods, GA implementation).

### Estimation of enzyme levels

Upon fixing the estimated elementary kinetic parameters (that is, *K*_*m*_), we next estimated 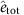 for the mutant strains growing anaerobically and those with pyruvate or acetate as the carbon substrate, separately. We note that this is equivalent to estimating *v*_max_ (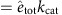) in Michaelis–Menten description, while fixing *K*_*m*_ to the estimated values (see Supplementary Methods, GA implementation). This is because we anticipated that only the enzyme levels are likely to change significantly when growth is switched from aerobic to anaerobic or to an alternate carbon substrate^61^,^62^,^63^. Therefore, the 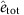 for all reactions in the model were allowed to vary from zero (that is, deletion) to a 10-fold overexpression (that is, 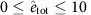) for mutant strains growing (i) anaerobically with glucose (that is, two flux data sets), (ii) aerobically with pyruvate as the carbon substrate (that is, three flux data sets) and (iii) aerobically with acetate (that is, one flux data set). The total number of enzymes whose total normalized pool was allowed to vary was kept at a minimum to avoid model overparameterization (see Supplementary Methods, enzyme level changes).

### Evaluation of predicted yields for overproducing mutants

We implemented enzyme level changes by allowing the total pool of the normalized enzyme to vary between a 10-fold downregulation and the wild-type level (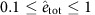) for reported enzyme downregulations. For enzyme upregulations, the normalized enzyme level was set between the wild-type level and a 10-fold upregulation (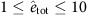). This approximation was used because the level of enzyme change is often not available in the relevant literature. Gene deletions were implemented by setting the 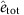 of the encoded enzyme to zero.

### Least squares model parameterization and yield analysis

The objective function of the parameterization problem *z* is to minimize the relative deviation of k-ecoli457 predicted flux *v*_*j*_ from the experimental data 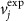 for reaction *j* with measured data *N* across all the mutant strains *M*. For each reaction with measured flux data, the average relative error is scaled by its coefficient of variation *CV*_*j*_ to capture the reported uncertainty in the experimental data (average scaled relative deviation)^64^. As a result, the reactions with tighter confidence intervals have a larger contribution in the objective function.


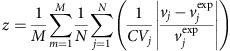


Product yield *y* is defined as the rate of reaction that produces the product per rate of uptake reaction (product mol per glucose mol).


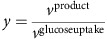


For comparisons, the majority of experimental studies reported only the measured product yield with no confidence range. Consequently, for each product yield the error was calculated based on relative deviation between the measured *y*^*exp*^ and predicted *y*^*pre*^ values.


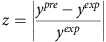


All parameterization problems were solved using a GA implementation. All calculations, including the ensemble construction and the GA problems, were implemented in MATLAB (MathWorks Inc.) and solved in parallel on three nodes of Intel Xeon E5-2670 v2 with 2.5 GHz (20 cores per node and 256 GB RAM) on the Penn State Lion-X clusters. The k-ecoli457 parameterization took ∼58,800 CPU-hour. Integrating k-ecoli457 (that is, solving the system of ordinary differential equations) for a given metabolic condition also takes up to a few minutes. The estimated elementary kinetic parameters as well as stoichiometry matrix of the model are available in Supplementary Data 1 and the executable k-ecoli457 is available for download in and at http://www.maranasgroup.com as a MAT-file (MathWorks Inc.).

### Code availability

The authors declare that the code supporting the findings of this study is available within the article's Supplementary Information files () and at http://www.maranasgroup.com as a MAT-file (MathWorks Inc.).

### Data availability

All data generated or analysed during this study are included in this published article and its Supplementary Information files.

## Additional information

**How to cite this article:** Khodayari, A. & Maranas, C. D. A genome-scale *Escherichia coli* kinetic metabolic model k-ecoli457 satisfying flux data for multiple mutant strains. *Nat. Commun.* **7,** 13806 doi: 10.1038/ncomms13806 (2016).

**Publisher's note:** Springer Nature remains neutral with regard to jurisdictional claims in published maps and institutional affiliations.

## Supplementary Material

Supplementary Information Supplementary methods, supplementary tables, supplementary figures, supplementary references.

Supplementary Data 1 List of metabolites, reactions and regulatory interactions

Supplementary Data 2 Estimated kinetic parameters and predicted metabolic fluxes, metabolite concentrations and product yields

Supplementary Software 1 MATLAB version of the model
