# Supplementary material for: A genome-scale Escherichia coli kinetic metabolic model k-ecoli457 satisfying flux data for multiple mutant strains
Source: Nat Commun. 2016 Dec 20;7:13806. doi: 10.1038/ncomms13806 (PMC5187423; doi:10.1038/ncomms13806)
Supplement: Supplementary Information — Supplementary methods, supplementary tables, supplementary figures, supplementary references. [file ncomms13806-s1.pdf]

Supplementary Methods

Estimation of model parameters satisfying experimental data for the wild-type and mutant strains

For k-ecoli457 development, we first constructed a genome-scale metabolic model of *E. coli* metabolism including all flux carrying pathways under the experimental conditions. The model includes 457 reactions and 337 metabolites. We also extracted 295 substrate level regulatory interactions from BRENDA [1] and EcoCyc [2] and integrated them into k-ecoli457. In addition, we defined a simplified version of biomass reaction described in [3] including all its constituent precursors. Next, we decomposed all metabolic and regulatory reactions into their elementary steps according to an iso-ordered mechanism [4]. This reaction decomposition expanded the network to 5,239 elementary interactions and 3,003 metabolites and enzyme complexes. We used the flux distribution of a wild-type strain grown aerobically with glucose [5] as the reference strain and generated an ensemble of 2^{17}=131,072 models, wherein all of them converge to the same flux distribution (see Figure 2a). Similar to the previous effort [6], the intracellular reactions carrying a zero flux in the reference strain but a non-zero flux in at least one mutant strain under aerobic glucose conditions are adjusted to carry a minimal amount of flux (i.e., equal to 0.05 mmol $gDW^{-1}h^{-1}$ per 100 mmol $gDW^{-1}h^{-1}$ of glucose uptake) in the reference strain to ensure the participation of the reaction in the construction of the ensemble. For example, the reactions catalyzed by 6-phosphogluconate dehydratase (EDD) and 2-dehydro-3-deoxy-phosphogluconate aldolase (EDA) do not carry flux in the wild-type strain but become active in Δ*pgi* [5]. For the reactions that carry a zero flux in the reference strain but a non-zero flux in at least one mutant strain under other growth

conditions (i.e., anaerobic growth or an alternate carbon substrate), we adjusted the flux of the reaction to carry a maximum amount (i.e., equal to 100 mmol gDW^{-1} h^{-1} per 100 mmol gDW^{-1} h^{-1} of glucose uptake) in the reference strain while fixing the total pool of the normalized enzyme level $\tilde{e}_{tot}$ to zero (i.e., $\hat{e}_{tot} = \sum \hat{e} = 0$). We allowed $\hat{e}_{tot}$ of those reactions to vary from zero (i.e., deletion) to a ten-fold over expression (i.e., $0 \leq \hat{e}_{tot} \leq 10$) under other growth conditions. This ensures that the reaction is inactive in the reference strain but can become active under other growth conditions. Example includes the reaction catalyzed by pyruvate formate lyase (PFL) which is not active under aerobic conditions but become active under fermentative (anaerobic) conditions. Estimation of the k-ecoli457 parameters was carried out using experimentally measured flux data for 25 mutant strains including 21 mutant strains grown with glucose (nineteen under aerobic [5] and two under anaerobic conditions [7]), three mutant strains grown with pyruvate under aerobic conditions [8] and one strain grown with acetate under aerobic conditions [9]. k-ecoli457 parameterization was proposed by first estimating equivalent Michaelis constants K_m using all the mutant strains data along with estimating equivalent maximal velocities v_{max} under each mutant condition, separately. Due to the computational complexity of the problem and the large number of parameters, we converted the parameterization problem into a two-step hierarchical optimization procedure. In the first step, the equivalent K_m and v_{max} values were estimated using the experimentally measured flux data for the single knockout mutants grown aerobically with glucose (i.e., a total of nineteen flux datasets). In addition, for the reactions catalyzed by isozymes, we estimated the lost activity upon deletion of one of the isozymes (see online Methods). In the second step, the estimated equivalent K_m values were fixed and v_{max} values were

estimated again for the strains grown anaerobically with glucose and those with alternate carbon substrates pyruvate and acetate, separately (i.e., a total of six flux datasets). The mathematical representation of the optimization problem is described below.

(a) Estimation of elementary kinetic parameters and isozymes activity using experimental data under aerobic conditions

The first step of the optimization problem requires definition of the following sets:

$I = \{metabolite, enzyme\ and\ enzyme\ complex\}$

$J = \{reaction\}$

$E_J = \{enzyme\ and\ enzyme\ complex\ of\ reaction\ j\}$

$L_J = \{elementary\ step\ of\ reaction\ j\}$

$MA = \{mutant\ data\ grown\ aerobically\ with\ glucose\}$

$N = \{reaction\ with\ available\ flux\ measurements\}$

$$RIS_J = \begin{Bmatrix} reaction\ catalyzed\ by\ isozymes\ with\ experimentally \\ measured\ flux\ data\ for\ one\ of\ its\ isozymes\ deletion \end{Bmatrix}$$

$CIS_J = \{isozyme\ of\ reaction\ j\}$

$P = \{models\ in\ the\ ensemble\}$

We also define the following variables and parameters:

Variables:

$$y_{jp} = \begin{cases} 1, & if\ kinetic\ parameter\ of\ reaction\ j \in J\ is\ selected \\ & from\ model\ p \in P\ in\ the\ ensemble \\ 0, & otherwise \end{cases}$$

$\kappa_{jp}^{2l_j-1}$= Sampled elementary kinetic parameters of the model $p \in P$ in the ensemble for the forward elementary mechanism of reaction $j \in J$ in step $l_j \in L_J$

$\kappa_{jp}^{2l_j}$= Sampled elementary kinetic parameters of the model $p \in P$ in the ensemble for the reverse elementary mechanism of reaction $j \in J$ in step $l_j \in L_J$

$k_j^{2l_j-1}$= Forward kinetic parameter of elementary reaction $j \in J$ in step $l_j \in L_J$

$k_j^{2l_j}$= Reverse kinetic parameter of elementary reaction $j \in J$ in step $l_j \in L_J$

v_j= Flux of reaction j

$\tilde{v}_j^{2l_j-1}$= Forward flux of the elementary reaction $j \in J$ in step $l_j \in L_J$

$\tilde{v}_j^{2l_j}$= Reverse flux of the elementary reaction $j \in J$ in step $l_j \in L_J$

$\hat{c}_i$= Normalized concentration of metabolite $i \in I$

$\hat{e}_i$= Fraction of free enzyme or enzyme complex $i \in I$

$\alpha_j^{cis_j}$= Activity loss of isozymes catalyzing reaction $j \in J$ upon deletion of cis_j

Parameters:

$\tilde{S}_{i,j}$= Stoichiometric coefficient of reaction $j \in J$ and metabolite $i \in I$ after reaction decomposition based on the elementary mechanism

v_j^{exp} = Experimental measurements of flux of reaction $j \in J$ in different mutant strains

CV_j = Coefficient of variation for reaction $j \in J$ with available experimental measurements in the reference strain (wild-type)

$ris_j = \begin{Bmatrix} reaction\ catalyzed\ by\ isozymes\ with\ experimentally \\ measured\ flux\ data\ for\ one\ of\ its\ isozymes\ deletion \end{Bmatrix}$

cis_j= Isozymes of reaction $j \in J$

The objective function of the optimization problem minimizes the average relative deviation between k-ecoli457 predictions and the experimentally measured flux datasets across all the nineteen mutant strains MA. For each reaction with measured flux data, the average relative error is scaled by its coefficient of variation to capture the reported uncertainty in the experimental data. As a result, the reactions with tighter confidence interval have a larger contribution in the objective function. The optimization problem is described as follows:

$$\underset{y_{jp}}{minimize} \quad z = \frac{1}{card(MA)} \sum_{m \in MA} \frac{1}{card(N)} \sum_{j \in N} \left(\frac{1}{CV_j} \left| \frac{v_j - v_j^{exp.}}{v_j^{exp.}} \right| \right)$$

subject to:

$$\left.\begin{array}{lll}
\sum_{j \in J} \tilde{S}_{i,j}.\tilde{v}_j = 0 & \forall i \in I & (1) \\
k_j^{2l_j-1} = \sum_{p \in P} y_{jp}\, \kappa_{jp}^{2l_j-1} & \forall j \in J, l_j \in L_J & (2) \\
k_j^{2l_j} = \sum_{p \in P} y_{jp}\, \kappa_{jp}^{2l_j} & \forall j \in J, l_j \in L_J & (3) \\
\sum_{p \in P} y_{jp} = 1 & \forall j \in J & (4) \\
\tilde{v}_j^{2l_j-1} = k_j^{2l_j-1} \left(\prod_{\tilde{S}_{i,j} < 0} \hat{c}_i^{|\tilde{S}_{i,j}|} \right) \left(\prod_{\tilde{S}_{i,j} < 0} \hat{e}_i \right) & \forall j \in J, l_j \in L_J & (5) \\
\tilde{v}_j^{2l_j} = k_j^{2l_j} \left(\prod_{\tilde{S}_{i,j} < 0} \hat{c}_i^{|\tilde{S}_{i,j}|} \right) \left(\prod_{\tilde{S}_{i,j} < 0} \hat{e}_i \right) & \forall j \in J, l_j \in L_J & (6) \\
v_j = \tilde{v}_j^{2l_j-1} - \tilde{v}_j^{2l_j} & \forall j \in J, l_j \in L_J & (7) \\
\hat{e}_{tot} = \sum_{i \in E_j} \hat{e}_i = 0 & \forall j \in \{Perturbed\ rxn\ in\ MA\} \backslash RIS_J & (8) \\
\hat{e}_{tot} = \sum_{i \in E_j} \hat{e}_i = 1 - \alpha_j^{cis_j} & \forall j \in \{Perturbed\ rxn\ in\ MA\} \cap RIS_J,\ cis_j \in \{deleted\ isozyme\} & (9) \\
\sum_{cis_j \in CIS_J} \alpha_j^{cis_j} = 1 & \forall j \in RIS_J & (10)
\end{array}\right\} \forall m \in MA$$

$$y_{jp} \in \{0,1\} \qquad \forall j \in J, p \in P$$

$$0 \le \alpha_j^{cis_j} \le 1 \qquad \forall j \in RIS_J, cis_j \in CIS_J$$

Constraint (1) represents conservation of mass for each metabolite, enzyme and enzyme complex in the model following elementary decomposition. Constraint (2) and (3) assign a sampled elementary kinetic value in the ensemble to the elementary forward and

reverse kinetic parameters of reaction j in step l_j, denoted as $k_j^{2l_j-1}$ and $k_j^{2l_j}$, respectively. $\kappa_{jp}^{2l_j-1}$ and $\kappa_{jp}^{2l_j}$ represent forward and reverse elementary kinetic parameters of reaction j in step l_j, respectively, in model p in the ensemble. For each elementary kinetic parameter there are *P* alternatives in the initial ensemble. y_{jp} is a binary variable acting as a "switch" that ultimately assigns the sampled parameter values in model p to all the elementary kinetic parameters of reaction *j*. Constraint (4) ensures that only one value for each elementary kinetic parameter is selected from the models in the ensemble. Constraints (5) and (6) represent the mass action kinetics for the forward and reverse elementary reactions, respectively. Constraint (7) computes the net flux of reaction *j* (v_j) as the difference between the corresponding elementary forward and reverse reactions. The flux of the perturbed reactions in mutant *MA* which is not catalyzed by isozymes is set to zero in constraint (8). For the reactions catalyzed by isozymes, constraint (9) allows the total level of enzyme e_i to vary between its deletion (i.e., 0) and the wild-type level (i.e., 1) to capture the lost activity upon deletion of one of the isozymes. Constraint (10) ensures that the sum of the total level of the isozymes cis_j catalyzing reaction *j* is set to the wild-type level (i.e., one). This optimization problem identifies the best combination of the sampled parameters in the initial ensemble as well as activity of isozymes with measured flux data upon their deletions. The experimentally measured flux data for the nineteen single knockout mutant strains including twelve single isozyme deletions were integrated at this step. These mutant strains were all grown under aerobic conditions with glucose as the sole carbon substrate (i.e., △*pgi,* △*pykA,* △*pykF,* △*ppsA,* △*gnd,* △*zwf,* △*rpe,* △*pfkA,* △*pfkB,* △*fbaB,* △*gpmA,* △*gpmB,* △*pgl,* △*rpiA,* △*rpiB,* △*talA,* △*talB,* △*tktA* and △*tktB*) [5]. The optimization problem was solved by iteratively adding mutant strain

data one at a time and initializing the problem from the best solution obtained at the previous iteration.

(b) Estimation of enzyme levels using experimental data under environmental perturbations

The estimated parameters at the first step were fixed and the levels of the enzymes were estimated through solving the second step of the optimization procedure (see Materials and methods). Thus we defined the identified elementary kinetic parameters and the lost of activity as the parameters:

Parameters:

y_{jp} = Identified binary variables

$\alpha_j^{cis_j}$= Identified activity loss of isozymes catalyzing reaction $j \in J$ upon deletion of cis_j

$k_j^{2l_j-1}$= Identified forward kinetic parameter of elementary reaction $j \in J$ in step $l_j \in L_J$

$k_j^{2l_j}$= Identified reverse kinetic parameter of elementary reaction $j \in J$ in step $l_j \in L_J$

This problem also requires defining three additional subsets and one variable:

Subsets:

$$D = \begin{Bmatrix} mutant\ condition\ (anaerobic\ growth\ or\ pyruvate\ or\ acetate\ as \\ the\ carbon\ substrate) \end{Bmatrix}$$

$$MN = \begin{Bmatrix} mutant\ data\ grown\ anaerobically\ and\ those \\ with\ pyruvate\ or\ acetate\ as\ substrate \end{Bmatrix}$$

$$R_d = \{Reactions\ whose\ enzyme\ level\ was\ allowed\ to\ vary\ in\ mutant\ condition\ d\}$$

Variable:

β_j^d= The change in the enzyme level of reaction j in condition d

The experimentally measured flux data for the remaining six mutant strains MN under three different growth conditions D were integrated at this step to estimate the minimum number of enzymes whose level was allowed to vary under each condition, separately (i.e., a wild-type, Δzwf and Δgnd under aerobic conditions with pyruvate as the carbon substrate [8] and a wild-type strain grown under aerobic conditions with acetate as the carbon substrate [9]). The objective function of the second step of the optimization problem is still minimizing the average relative deviation between k-ecoli457 predictions and the experimentally measured flux datasets. The optimization problem is described as follows:

$$\underset{\beta_j^d}{minimize} \quad z = \frac{1}{card(MA)} \sum_{m \in MN} \frac{1}{card(N)} \sum_{j \in N} \left(\frac{1}{CV_j} \left| \frac{v_j - v_j^{exp.}}{v_j^{exp.}} \right| \right)$$

subject to:

$$\text{Constraints (1, 5-8)} \qquad \forall m \in MN$$

$$\sum_{i \in E_j} \hat{e}_i = \beta_j^d \qquad \forall d \in D, j \in R_D \quad (11)$$

$$0 \leq \beta_j^d \leq 10$$

The objective function and constraints (1, 5-8) are similar to those of the first step of the optimization problem. The binary variables y_{jp} as well as the elementary kinetic parameters $k_j^{2l_j-1}$ and $k_j^{2l_j}$ are obtained in the first step of the optimization problem, through identifying the best set of model parameters in the initial ensemble. In addition, for the reactions catalyzed by isozymes with available experimentally measured flux data of their deletion, the activity of each isozyme was estimated. Constraint (11) allows the

level of R enzymes to vary under anaerobic growth or alternate substrates D in order to capture metabolic transition.

Simplified biomass reaction

We integrated a simplified version of the biomass equation for *E. coli* described in [3], which acts as a drain for the amino acids as well as all the main constituent precursors in central metabolism. For the pathways absent in the simplified biomass equation, their main precursor in central metabolism was used and all unnecessary reactions were removed to make the model compact. For example, we defined an export reaction for Acetyl-CoA and G6P as a proxy for the lipid metabolism and nucleotide biosynthesis pathways drainage towards biomass.

0.205 G6P + 0.071 F6P + 0.754 R5P + 0.129 GAP + 0.619 3PG + 0.051 PEP + 0.083 PYR + 2.510 AcCoA + 0.087 AKG + 0.340 OAC + 0.488 Ala + 0.281 Arg + 0.229 Asn + 0.229 Asp + 0.087 Cys + 0.250 Glu + 0.250 Gln + 0.582 Gly + 0.090 His + 0.276 Ile + 0.428 Leu + 0.326 Lys + 0.146 Met + 0.176 Phe + 0.210 Pro + 0.205 Ser + 0.241 Thr + 0.054 Trp + 0.131 Tyr + 0.402 Val + 0.443 MEETHF + 33.247 ATP + 5.363 NADPH → 39.68 biomass + 1.455 NADH

GA implementation of the optimization problems

Both optimization problems were solved using a binary coded genetic algorithm (GA) implementation in MATLAB (MathWorks Inc.) as described below:

Genotype and phenotype of chromosomes: The problem solutions are represented as a population of chromosomes in the GA representation. Each chromosome is composed of a set of genes where each gene represents one problem variable in a discrete representation. Genotype of each gene is parsed to its phenotype by a decimal decoding operation. In the first step of the optimization problem, one integer variable is defined for each reaction j to assign a sampled set of elementary kinetic parameters from model p in

the ensemble, as represented by binary variables y_{jp} in the optimization formulation. In addition, for the reactions catalyzed by isozymes with available experimentally measured flux data for one of the isozymes deletions, the total enzyme level $\hat{e}_{tot}$ is defined as a continuous variable. We defined a chromosome to represent both the integer and continuous variables in the first step of the optimization problem (see Supplementary Figure 1.a). For the integer variables, each gene represents a sampled model out of the *P* model in the ensemble for each kinetic parameter (i.e., 1≤decimal(bin_a)+1≤number of sampled models in the ensemble *P,* where *bin* represents the binary value). The length of each gene (i.e., number of bits) is defined as $\lceil \log_2(number\ of\ models) \rceil$ (where $\lceil x \rceil$ represent the smallest integer not less than x), which is represented as a in Supplementary Figure 1.a. In order to prevent an undefined search space, the number of models in the ensemble was set to be an integer exponent of 2 (i.e., 2^{17}). We note that 2^{17} models have been sampled in the ensemble as no further improvement in the model predictions and convergence to the optimal solution were achieved for a larger ensemble size. Thus, the number of bits a for the integer variables was set to 17. Alternatively, for the continuous variables we defined a gene for each enzyme level of the perturbed reactions catalyzed by isozymes that can take a value between its deletion and the wild-type level (i.e., $0 \le \hat{e}_{tot} \le 1$) in a discrete representation. The reduced level of the enzyme upon deletion of one of the isozymes is obtained by mapping the scaled value between zero and one following decimal decoding (i.e., 0≤scaled value=decimal(bin_b)/(2^b-1)≤1, where *b* represents the number of bits for each gene). The length of each gene specifies the resolution of the solution space as 1/(2^b-1). Thus, for a desired resolution, the length of each gene is $\lceil \log_2(resolution + 1/_{resolution}) \rceil$. We set the number of bits b to 16 as no

improvement was observed for a higher resolution (i.e., equivalent of an approximate resolution of >1.5×10^{-5}). In the second step of the optimization problem, the level of the enzymes is defined as a variable under each mutant condition, separately. Similarly, we defined a gene for each enzyme level that can take a value between its deletion and a ten-fold up regulation (i.e., $0 \leq \hat{e}_{tot} \leq 10$) in a discrete representation under each mutant condition, separately. First, we mapped the binary value of each gene to a scaled value (i.e., 0≤scaled value=decimal(a_c)/(2^c-1)≤1, where c represents the length of each gene). We set the number of bits c to 16 (i.e., equivalent of an approximate resolution of >1.5×10^{-5}) as no improvement was observed for a higher resolution (see supplementary Figure 1.b). Next, the enzyme level under each mutant condition is obtained by mapping the scaled value between zero and a ten-fold overexpression (i.e., 0≤ scaled value×UB ≤10, where UB is the upper bound of the enzyme level, 10).

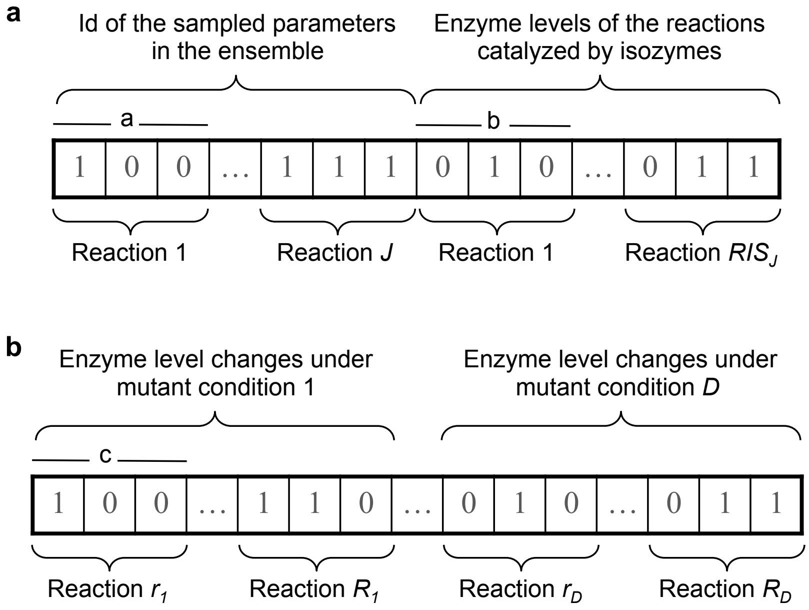

Supplementary Figure 1 Genotype and phenotype representations of each chromosome. (a) In the first step of the optimization problem, the chromosome includes both integer and continuous variables representing the id of the sampled model in the ensemble and

the reduced level of the enzyme for the perturbed reactions catalyzed by isozymes, respectively. The total number of metabolic reactions and the reactions catalyzed by isozymes with measured flux data of one of the isozyme deletion are represented by *j* and RIS_j, respectively. (b) In the second step of the optimization problem, the chromosome includes only continuous variables representing the change in the level of enzymes when growth is switched from aerobic glucose to the other growth conditions. Number of bits is represented by *a*, *b* and *c*. *D* represents the number of other growth conditions (i.e. not grown aerobically with glucose) and *R* represents the number of reactions whose level was allowed to vary under those conditions.

Population size: In all simulations the population size was set to four to six times the number of variables (i.e., genes) in the chromosomes.

Initialization of population: A uniform distribution was used to populate the chromosomes in the initial and remaining generations. Following the initial generation, only one chromosome was initialized with the best solution obtained in the previous generation.

Crossover operator: We examined algorithm performance with different crossover fractions to find an optimal value. The GA problem initialized with 0.8 crossover fraction until no improvement is observed in the fitness after 40 generations. Next, the problem is run again with a more conservative crossover fraction (i.e., 0.9) until no improvement is observed again in the fitness after 40 generations. A scattered crossover was also used to construct the child chromosome by combining the parent's genes.

Termination criterion: The GA procedure is terminated when the fitness of the elite chromosome is considered sufficiently high (i.e., as good as the elite chromosome before the addition of new flux dataset) or if no improvement is observed in the elite chromosome after 40 generations. Overall, we observed that in the majority of runs, the fitness reaches the saturation level as the number of generations exceeds 100. However,

each run was submitted for another 40 generations to ensure there is no further improvement after 40 generations.

Predicted steady-state metabolic flux distribution

Following model parameterization, we observed that for 61% of the reactions the predicted flux data by k-ecoli457 are within the experimentally reported ranges (see supplementary Figure 2.a and Results section of the manuscript). We also tested the residual distribution of the predicted fluxes as a measure of statistical correctness of k-ecoli457 predictions (see supplementary Figure 2.b-c). In general, we observed that the distribution is approximately normal implying the statistical accuracy of k-ecoli457 predicted fluxes (see Supplementary Data 1 for the predicted flux distributions in all the mutant strains by k-ecoli457).

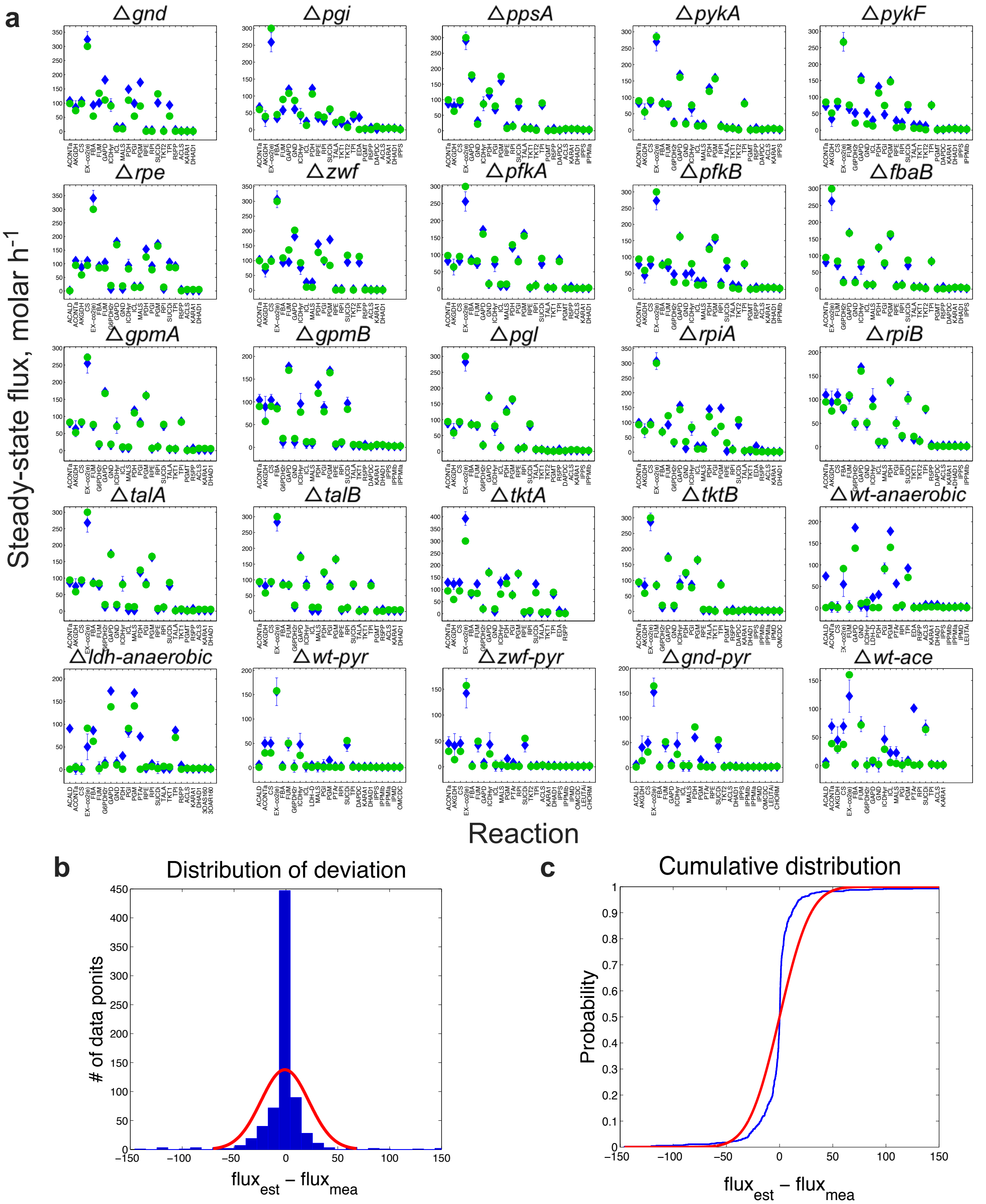

Supplementary Figure 2 (a) The experimentally measured and predicted steady-state fluxes by k-ecoli457 in the mutant strains for up to 25 reactions (see Supplementary Data 2 for a complete list of predicted fluxes). Blue diamonds represent the experimental measurements for each mutant and green circles represent the predicted flux distributions by k-ecoli457. Error bars denote one standard deviation confidence interval for the corresponding reaction in the wild-type strain. (b) The residual distribution between the predicted steady-state flux data by k-ecoli457 and measured values. Blue bars denote k-ecoli457 predictions and the red distribution shows the fitted distribution. (c) The cumulative distribution between the predicted steady-state flux data by k-ecoli457 and

measured values. The blue distribution shows k-ecoli457 predictions and red distribution shows the normal distribution.

Statistical significance of the model parameterization using a cross-validation analysis

In order to assess over-fitting of the data to the underlying model, we performed leave-one-out and leave-two-out cross validation tests by iteratively excluding, respectively, one and two mutant stains flux data at a time from the training set and then test the resulting model against the excluded data (the results of leave-two-out are not shown as they exhibited the same trends as leave-one-out tests). Overall, the cross validation analysis reflects the dependence of prediction on the inter-relation of the training datasets. The analysis displayed that for the mutant strains under aerobic conditions with glucose (nineteen mutants) and pyruvate (three mutants) as the carbon source, the model parameterization is generally robust to the excluded data with an average increase in average scaled deviation by 5% (15% increase was observed in the previously published core model [6]) across all validation tests (see Supplementary Figure 3). This is due to the fact that the integrated experimental data are comprised of a diverse set of enzyme perturbation strategies in different parts of the metabolism so that model parameterization is still supported by the data of the mutants located in the same vicinity of the cross validated mutant. For example, in the core model we reported a failure in cross-validation of Δ*pgi* due to the absence of flux data of the adjacent mutants for its model parameterization [6]. This issue is addressed here through integration of three additional sets of knockout mutants' flux data in the preparatory phase of glycolysis (i.e., Δ*pfkA*, Δ*pfkB* and Δ*fbaB*). The same holds true for the remaining eighteen cross validation tests with glucose as the carbon substrate including those in pay-off phase of

glycolysis (i.e., Δ*gpmA,* Δ*gpmB,* Δ*pykA,* Δ*pykF* and Δ*ppsA*) as well as oxidative (i.e., Δ*zwf,* Δ*pgl* and Δ*gnd*) and non-oxidative (i.e., Δ*rpe,* Δ*rpiA,* Δ*rpiB,* Δ*talA,* Δ*talB,* Δ*tktA* and Δ*tktB*) sections of the PP pathways. Likewise, a robust model parameterization was observed during cross validation of three mutant data with pyruvate as the carbon substrate (i.e., the wild-type, Δ*zwf* and Δ*gnd*), as their parameterizations were still endorsed by two other mutant data. For the latter case with the exceptions of the reactions catalyzed by *pgi*, *zwf* and *gnd*, we note that the observed robust cross validation of the wild-type strain is a result of the similarity of the measured flux data compared to two other mutants. As expected, this robust model parameterization was not the case for cross validation tests of the two mutant strains under anaerobic conditions with glucose as the carbon source (i.e., the wild-type and Δ*ldh*) as alluded by higher prediction deviations from experimental data (i.e., a 14% increase in average scaled deviation). In particular, the measured flux data of the fermentative products (i.e., formate, lactate, acetate and ethanol) are significantly different in both mutant strains. This variation in the activity of the fermentation pathways was not considered during each mutant cross validation test and thereby the predicted enzyme levels could not properly capture the excluded phenotypes. Even though these discrepancies propagate to some extent in other part of the network, the model prediction is still acceptable for the remaining reactions (i.e., an average 8% deviation from the experimental ranges). We note that due to having only one strain data grown on acetate, we did not perform a cross validation test for this strain and thus no conclusion was drawn for the robustness of the estimated parameters.

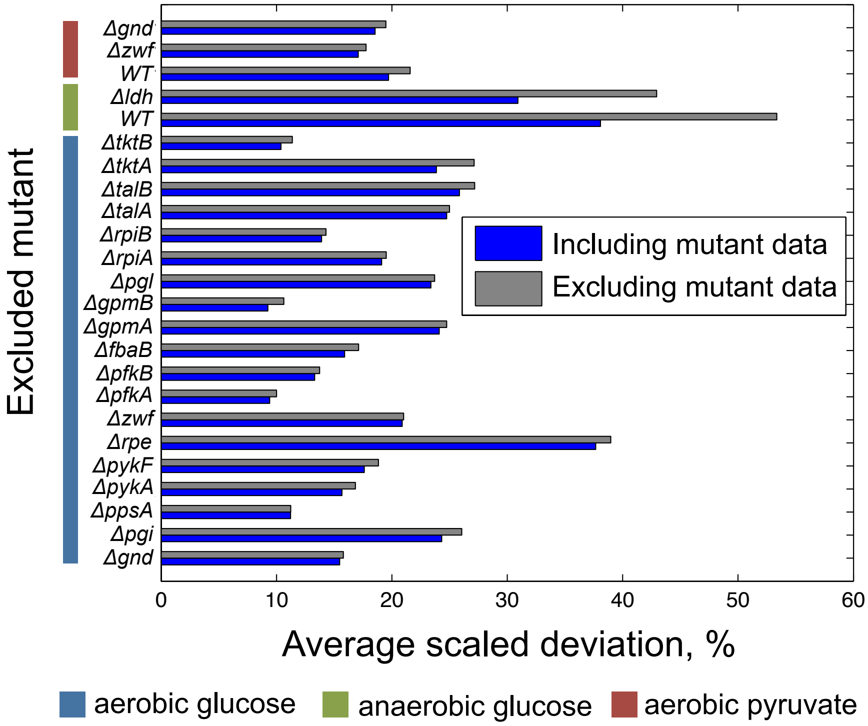

Supplementary Figure 3 Cross-validation analysis. Gray bars represent the average scaled deviation of the predicted steady-state fluxes for different mutant strains upon their exclusion from the training dataset. Blue bars correspond to the average scaled deviation of the predicted steady-state flux distribution from the experimental measurements while including all mutant strains data. The difference between the two bars represents the increase in average scaled deviation upon excluding the flux data of the mutant strain.

Confidence intervals of the estimated and measured parameters

The predicted normalized metabolite concentrations and Michaelis-Menten constants are scaled by the corresponding metabolite concentration ranges in the reference (wild-type) strain in order to convert them into the actual ranges. As a result, large confidence errors in the experimental data for metabolite concentrations lead to large confidence intervals for model predictions (see Figures 3 and 4). We extracted five additional measured concentration datasets for the wild-type strain and used their intersection as the base confidence ranges to reduce the uncertainties of the base concentrations, as described in the Methods section (see Supplementary Table 1 and 2). In order to assess statistical

correctness of k-ecoli457 model predictions we performed a statistical analysis on the residual distribution of the predicted parameters. Overall, the results revealed a near-normal residual distribution implying statistical significance of the estimated parameters [10] (see Supplementary Figure 4).

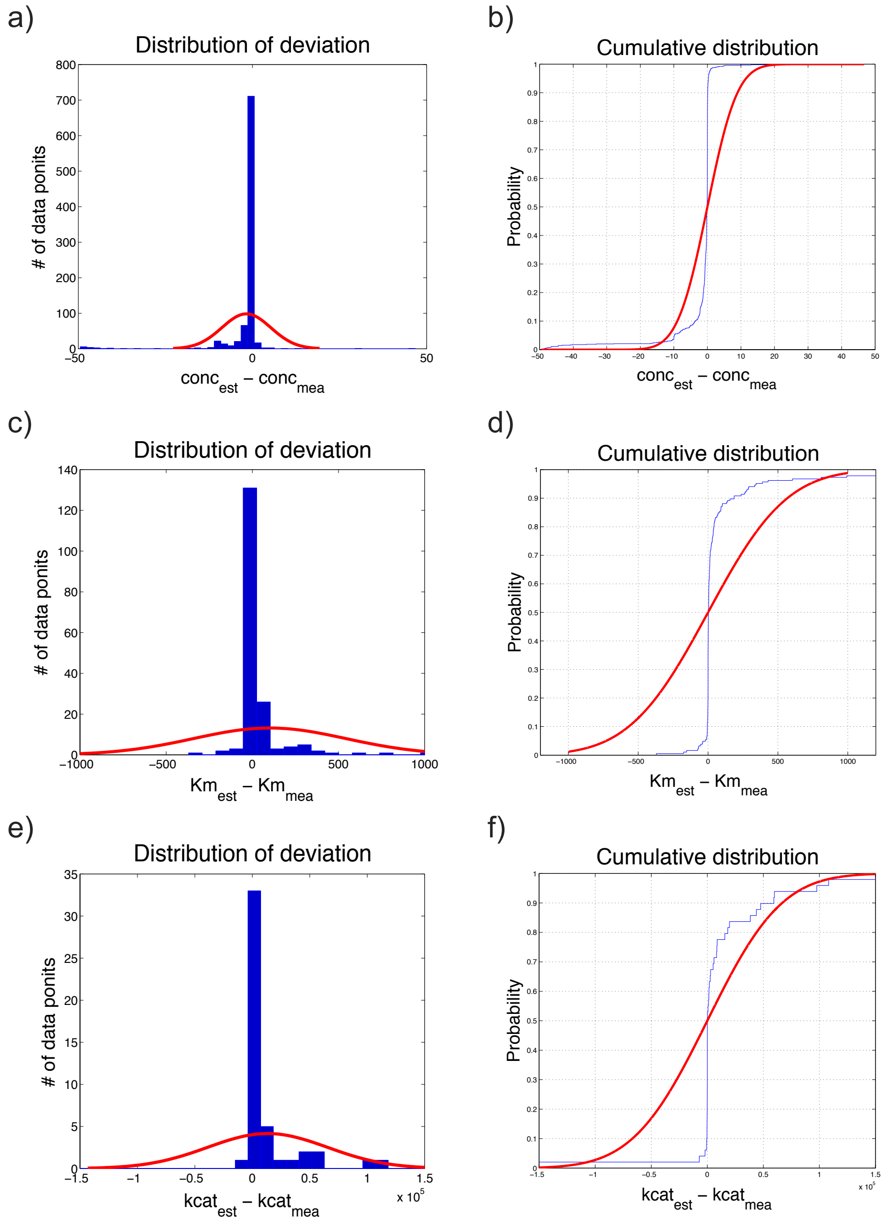

Supplementary Figure 4 The residual distribution and cumulative distribution between the measured and predicted (a-b) steady-state metabolite concentrations in the twenty mutant strains, and (c-d) K_m and (e-f) k_{cat} values by k-ecoli457. Blue bars denote k-

ecoli457 predictions and red curve shows the fitted distribution. The blue distribution shows k-ecoli457 predictions and the red distribution shows the normal distribution.

Supplementary Table 1 The list of flux datasets used for the k-ecoli457 parameterization

#	Genetic background	Strain	Growth condition	Substrate	Dilution rate (or specific growth rate), h^{-1}	Ref.
1	*wild-type*	BW25113	aerobic	glucose	0.2	[5]
2	Δ*pgi*	KO04				
3	Δ*pykA*	KO12				
4	Δ*pykF*	KO13				
5	Δ*ppsA*	KO14				
6	Δ*gnd*	KO17				
7	Δ*zwf*	KO15				
8	Δ*rpe*	KO18				
9	Δ*pfkA*	KO05				
10	Δ*pfkB*	KO06				
11	Δ*fbaB*	KO08				
12	Δ*gpmA*	KO10				
13	Δ*gpmB*	KO11				
14	Δ*pgl*	KO16				
15	Δ*rpiA*	KO19				
16	Δ*rpiB*	KO20				
17	Δ*talA*	KO23				
18	Δ*talB*	KO24				
19	Δ*tktA*	KO21				
20	Δ*tktB*	KO22				
21	*wild-type*	BW25113	anaerobic	glucose	0.16	[7]
22	Δ*ldh*	JW1375			0.14	
23	*wild-type*	BW25113	aerobic	pyruvate	0.2	[8]
24	Δ*zwf*	JWK1841				
25	Δ*gnd*	JWK2011				
26	*wild-type*	K12	aerobic	acetate	0.22	[9]

Supplementary Table 2 The list of metabolite concentration datasets used for the k-ecoli457 testing

#	Genetic background	Strain	Growth condition	Substrate	Dilution rate (or specific growth rate), h^{-1}	Ref.
1	*wild-type*	BW25113	aerobic	glucose	0.2	[5]
2	Δ*pgi*	KO04				
3	Δ*pykA*	KO12				
4	Δ*pykF*	KO13				
5	Δ*ppsA*	KO14				
6	Δ*gnd*	KO17				
7	Δ*zwf*	KO15				
8	Δ*rpe*	KO18				

9	Δ*pfkA*	KO05				
10	Δ*pfkB*	KO06				
11	Δ*fbaB*	KO08				
12	Δ*gpmA*	KO10				
13	Δ*gpmB*	KO11				
14	Δ*pgl*	KO16				
15	Δ*rpiA*	KO19				
16	Δ*rpiB*	KO20				
17	Δ*talA*	KO23				
18	Δ*talB*	KO24				
19	Δ*tktA*	KO21				
20	Δ*tktB*	KO22				
21	*wild-type*	K12	aerobic	acetate	0.22	[17]
22	*wild-type*	BW25113	aerobic	glucose	0.2	[11]
23		BW25113			0.52	[12]
24		W3110			0.1, 0.32, 0.55	[13]
25		BW25113			0.1	[14]
26		BW25113			0.2	[15]
27		W3110			0.1	[16]
28		NCM3722				[17]

Correlation between measured and predicted product yields

The Pearson correlation coefficient is used to represent the strength of a linear association between the experimentally measured product yields $y^{exp.}$ and predicted values $y^{pre.}$ by k-ecoli457 ($\bar{y}$ shows the mean value). A Student's t distribution is used to transform the correlation (see Supplementary Table 3 for Pearson correlation coefficients of predicted and measured product yield values).

$$r = \frac{\sum_z \left(y_z^{exp} - \bar{y}^{exp}\right)\left(y_z^{pre} - \bar{y}^{pre}\right)}{\sqrt{\sum_z \left(y_z^{exp} - \bar{y}^{exp}\right)^2} \sqrt{\sum_z \left(y_z^{pre} - \bar{y}^{pre}\right)^2}}, z = \{measured\ product\ yield\}$$

Supplementary Table 3 Pearson correlation coefficient for the predicted and measured product yields

	k-ecoli457	FBA	MOMA	Max. yield
Measured	0.8388<br>$P < 10^{-4}$	0.1826<br>$P < 10^{-3}$	0.3707<br>$P < 10^{-4}$	0.4650<br>$P < 10^{-4}$
k-ecoli457		0.0721<br>$P < 0.1958$	0.2518<br>$P < 10^{-4}$	0.4225<br>$P < 10^{-4}$

FBA		0.5978<br>$P < 10^{-4}$	0.2462<br>$P < 10^{-4}$
MOMA			0.2477<br>$P < 10^{-4}$

k-ecoli457 parameterization under anaerobic growth or alternate substrates

Regulatory programs at the transcription and (post)translation levels are the main cellular strategy to optimally modulate enzyme abundances under different conditions (e.g., growth mode or substrate changes) [18, 19]. In general, these regulatory events act by up-regulating key enzymes with originally low basal expressions [20] and repressing enzymes that interrupt cell growth [21] through subtle regulation mostly acting at branch points in central metabolism [22]. In order to capture these regulatory effects and parameterize k-ecoli457 under anaerobic growth or alternate substrates, we used the parameters estimated using flux data of the mutant strains grown aerobically with glucose. We fixed the estimated parameters (i.e., K_m) but allowed the level of a limited number of enzymes (i.e., v_{max} in Michaelis-Menten description) to vary from zero (i.e., deletion) to a ten-fold up-regulation (i.e., $0 \leq \hat{e}_{tot} \leq 10$) to match the measured flux datasets under each mutant condition independently. These conditions include (i) anaerobic growth and (ii) pyruvate or (iii) acetate as the carbon substrate.

In general, we observed that k-ecoli457 parameterization performed well under anaerobic growth and the two other substrates by accurately capturing the metabolic regulations through the estimated enzyme level changes. For example, the metabolic transition from aerobic to anaerobic conditions is mainly controlled at the transcription level by the two-component Arc system (aerobic respiratory control) [23] and FNR (fumarate and nitrate reductase regulation) [24]. During the transition, they repress the PP pathway [25] and

the TCA cycle [26] while activate glycolysis [7] and fermentative pathways [26]. We observed that the estimated enzyme levels accurately captured these metabolic phenotypes by changing the level of 81 enzymes in order to satisfy the measured flux datasets. For example, under anaerobic conditions, the electron transport chain in the oxidative phosphorylation pathway is not active, thus preventing the oxidation of cofactor nadh, which is generated instead in the pay-off phase of glycolysis by glyceraldehyde 3-phosphate dehydrogenase (GAPD). Without an adequate nadh sink, a significant amount of metabolic flux is directed towards ethanol, acetate, lactate and formate to maintain redox balance and cellular growth. This increased activity of fermentative products was adequately captured by the estimated up-regulation of acetaldehyde dehydrogenase (ACALD) (i.e., nine-fold), phosphotransacetylase (PTAr) (i.e., seven-fold), lactate (i.e., six-fold) and formate transport reactions (i.e., five-fold). In addition, the oxidative part of the TCA cycle is repressed by ArcA inhibition [27] while the reductive (i.e., C_4) section is activated by FNR [28] to convert anaplerotic flux towards succinate, malate and fumarate. This branching of the TCA cycle was also captured by down-regulating aconitase (ACONTa) (i.e., ten-fold) in oxidative section and up-regulation of fumarase (FUM) (i.e., eight-fold) and malate dehydrogenase (MDH) (i.e., nine-fold) in the reductive section. The increased activity of glycolysis and reduced activity of the PP pathways were also captured by up-regulating pyruvate kinase (PYK) (i.e., eight-fold) and phosphofructokinase (PFK) (i.e., eight-fold) and down-regulating ribose transport (i.e., ten-fold) and transaldolase (TALA) (i.e., two-fold).

The same agreement with the experimental reports was also observed for the mutant strains with pyruvate or acetate as the carbon substrate by changing 35 and 5 enzyme

levels, respectively. For the strains grown on pyruvate, activity of the PP pathway is repressed, thus decreasing the level of reducing power (i.e., nadph) required for cell biosynthesis. In general, up-regulating the TCA cycle and the anaplerotic pathway [8] as well as transhydrogenase (THD) [29], which equilibrates the nadh and nadph pools, are two strategies to fulfill the nadph demand in *E. coli*. We observed that the estimated enzyme levels predicted the increased activity of the TCA cycle and the anaplerotic pathway to produce cytosolic nadph and replenish the C_4 intermediate in the TCA cycle by up-regulating ACONTa (i.e., two-fold), succinyl-coa synthetase (SUCOAS) (i.e., four-fold) and FUM (i.e., nine-fold). However, we note that the estimated enzyme levels could not capture the increased activity of THD that is possibly due to the lack of training dataset representing such phenotype. The experimentally reported increase in the carbon dioxide evolution rate [8] was also captured by up-regulating carbon dioxide transport (i.e., eight-fold). For the strain grown on acetate, the PP pathway contributes negligibly to the metabolism, as a large fraction of acetate is routed towards the TCA cycle thus satisfying nadph demands for cell growth [9]. The estimated enzyme levels suggested a deviation of gluconeogenesis flux from glyceraldehyde 3-phosphate (g3p) towards serine to reduce the activity of the PP pathway by up-regulating phosphoserine phosphatase (PSP) (i.e., seven-fold).

While the above described estimated enzyme level changes recapitulated mostly the known regulatory effects, the initially integrated regulatory events at substrate-level in k-ecoli457 also simultaneously reapportioned the enzyme levels to reproduce the training datasets in each mutant strain. For example, atp is a competitive inhibitor of PYK [30] and PTAr [31], therefore the degree of repression imposed on the activity of glycolysis

and the fermentative pathways is proportional to its concentration. The predicted lower concentration of atp under anaerobic compared to that of aerobic conditions (i.e., 1.3-fold) reduced this regulatory effect as reported experimentally [13, 17]. Likewise, the reduced inhibitory effects of malate on phosphoenolpyruvate carboxylase (PPC) [32] increased the anaplerotic flux towards the reductive section of the TCA cycle through the malate shunt [13, 17]. In addition, the experimentally reported acetyl-coa overflow in Δ*ldh* [7] was partially captured by up-regulating (i.e., 1.3-fold) malonyl-coa-acp transacylase (MCOATA) due to the reduced inhibition of coa [33], thus directing acetyl-coa towards membrane lipid metabolism. The substrate-level regulations also led to accurate predictions of metabolic regulation during growth with the two other carbon substrates. For example, for the strains grown on pyruvate, the predicted reduced inhibition of phosphate resulted in up-regulating (i.e., 1.2 fold) fructose-bisphosphatase (FBP) [34] and thus captured the increased activity of phosphoglucose isomerase (PGI), as reported experimentally [8]. For the strain grown on acetate, the increased activity of the TCA cycle and the glyoxylate shunt [9] was also estimated by reduced inhibition of phosphoenolpyruvate, thus up-regulating ICDH (i.e., 1.1-fold) and ICL (i.e., 1.7-fold) [35], respectively. While the activity of the PP pathway was repressed by the estimated total enzyme level changes, up-regulating (i.e., 1.1-fold) transaldolase (TALA) was also suggested to ensure a minimal production of erythrose 4-phosphate (e4p) for the biosynthesis of nucleotides and aromatic amino acids [9]. Overall, these observations indicated that k-ecoli457 parameterization led to accurate phenotype predictions even when growth mode or carbon substrate was changed by concurrently capturing the effect of all regulatory events. While regulatory interactions at transcription and

(post)translation level are often responsible for drastic enzyme activity changes mainly at key branch points of metabolism [18, 19], regulations at substrate-level modulate the enzyme activity by sensing the metabolic level and inducing appropriate regulatory signals [22]. This implementation of regulation enabled us to efficiently integrate available measured fluxes for different genetic variants under various growth conditions to parameterize a single model.

Statistical analysis of the estimated product yield

We analyzed the residual distribution of the predicted product yields as a measure of statistical correctness of model predictions (see Supplementary Figure 5). In general, we observed that the distribution is approximately normal implying the statistical accuracy of k-ecoli457 predictions and systematic errors in the FBA, MOMA and maximization of product yield predictions.

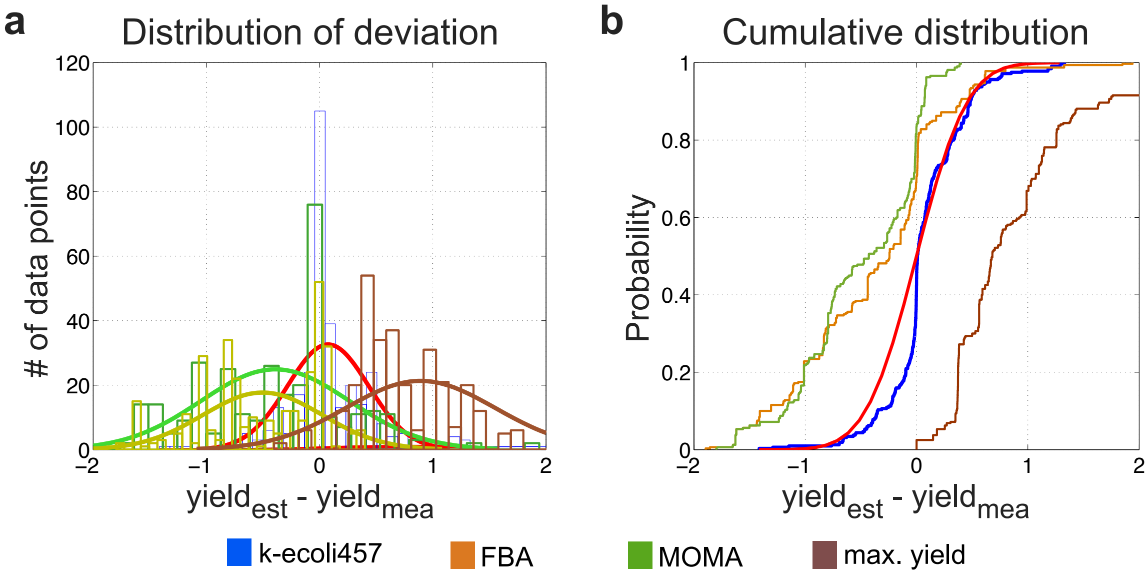

Supplementary Figure 5 (a) The residual distribution between the predicted product yields by k-ecoli457, FBA, MOMA and maximization of product yield, and experimentally measured values. The fitted distributions are shown for each model. (b) The cumulative distribution between the predicted product yields by k-ecoli457, FBA,

MOMA and maximization of product yield, and experimentally measured values. The normal distribution is shown in red.

Enzyme level changes under substrate change or growth condition

We estimate the enzyme level changes when we switch the growth mode from aerobic to anaerobic conditions and also carbon substrate from glucose to pyruvate or acetate. This is because regulatory events at the transcription and (post)translation levels are the main cellular strategy to optimally modulate enzyme abundances under such perturbation scenarios [18, 19, 22]. For example, the reaction catalyzed by pyruvate formate lyase (PFL) does not carry any flux under aerobic conditions but becomes active under anaerobic conditions. Likewise, the reactions in fermentative pathways carry higher flux under anaerobic conditions to maintain redox balance for cell growth. In order to capture these regulations, we allowed the level of a limited number of enzymes R_D under each mutant condition D (i.e., v_{max} in Michaelis-Menten description) to vary between 0 and a 10-fold over expression (i.e., $0 \leq \hat{e}_{tot} \leq 10$). In order to identify the minimum number of enzymes of which altered activity is required to reproduce the anaerobic data as well growth with pyruvate or acetate, we developed trade-off plots for each mutant condition, separately, and used the values in the “break points” as the optimal number of enzyme levels with varied levels (see Supplementary Figure 6).

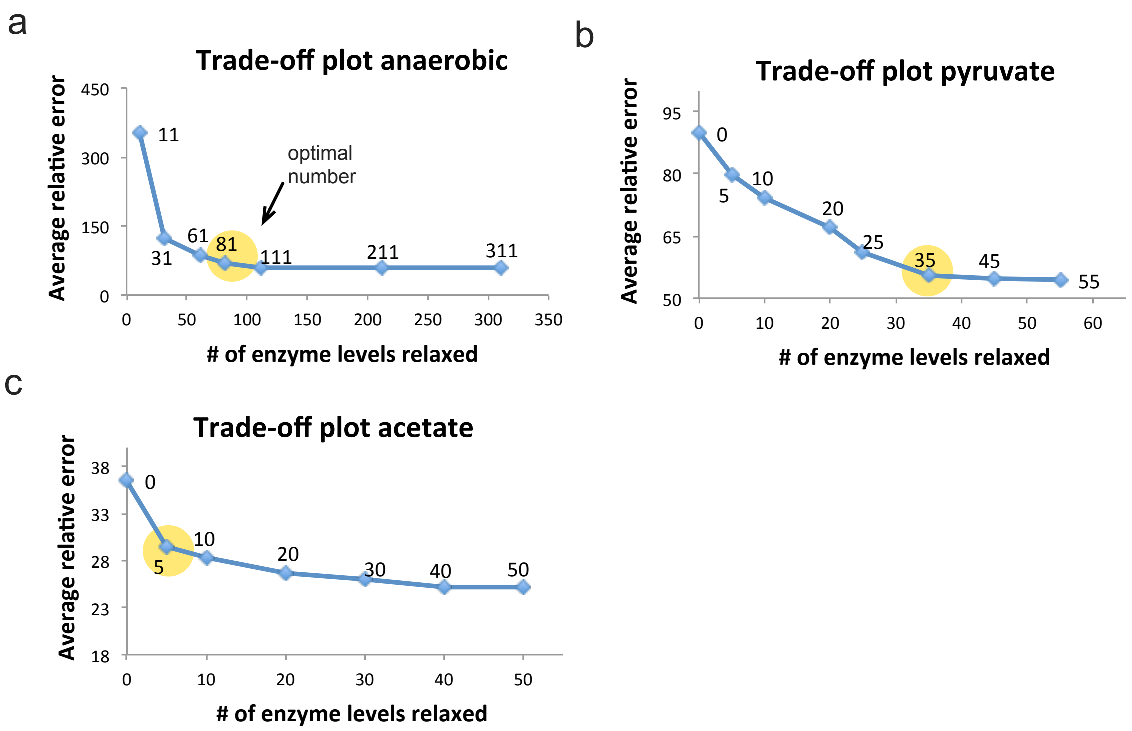

Supplementary Figure 6 Trade-off plots to identify a minimum number of enzymes whose levels were allowed to vary under (a) anaerobic conditions, (b) pyruvate and (c) acetate as the carbon substrates. The highlighted numbers were used as the minimum number of enzymes with altered levels.

Conversion of the estimated elementary kinetic parameters to Michaelis-Menten constants

Elementary kinetic is the most fundamental kinetic description at molecular level in which reactions are decomposed into their elementary steps. For enzymatic reactions, reactants first bind to the enzyme one at each step to form a complex. Reaction transformation (e.g., isomerization) takes place during conversion of the central complexes and subsequently products are released one at each step. The order of binding reactants to the complex or release of products from the complex depends on the reaction mechanism [4]. For example, for a reversible reaction with one substrate and one product with an iso-ordered mechanism (iso-ordered Uni-Uni):

$$S \overset{E}{\leftrightarrow} P$$

Elementary steps are:

$$E \quad + \quad S \underset{k_{-1}}{\overset{k_1}{\rightleftarrows}} ES \underset{k_{-2}}{\overset{k_2}{\rightleftarrows}} EP \underset{k_{-3}}{\overset{k_3}{\rightleftarrows}} E+P$$

$$e \qquad s \qquad\qquad x \qquad\qquad y \qquad\qquad p$$

where,

s= concentration of substrate S
p= concentration of product P
x= level of enzyme complex ES
y= level of enzyme complex EP
e= level of enzyme E

Next, we develop a mass action kinetics equation for each elementary reaction.

$$\tilde{v}_1 = k_1.s.e$$

where e can be expressed in terms of level of enzyme complexes and the initial level of enzyme E, e_{tot}:

$$e = e_{tot} - x - y$$

The elementary kinetic parameters can then be converted to their equivalent Michaelis-Menten constants by developing a quasi steady-state assumption for the reaction intermediates. For the enzyme complexes with a quasi steady-state assumption we can have:

$$\frac{dx}{dt} = yk_{-2} + (e_{tot} - x - y)sk_1 - (k_2 + k_{-1})x = 0$$

$$\frac{dy}{dt} = xk_2 + (e_{tot} - x - y)pk_{-3} - (k_3 + k_{-2})y = 0$$

This equation can be solved to substitute the enzyme concentration with the substrate and metabolite concentrations into the reaction rate:

$$\tilde{v} = k_3 y - k_{-3}(e_{tot} - x - y)p$$

Solving the system of equations gives (see Supplementary Table 4):

$$\tilde{v} = \frac{k_{cat}^f e_{tot} s - k_{cat}^b e_{tot} p}{1 + \frac{s}{K_m^S} + \frac{p}{K_m^P}} = \frac{v_{max}^f s - v_{max}^b p}{1 + \frac{s}{K_m^S} + \frac{p}{K_m^P}}$$

Supplementary Table 4 Elementary form of Michaelis-Menten Constant

Michaelis-Menten constant	Elementary constant
K_m^S	$\frac{k_{-1}k_{-2} + k_{-1}k_3 + k_2k_3}{k_1(k_{-2} + k_2 + k_3)}$
K_m^P	$\frac{k_{-1}k_{-2} + k_{-1}k_3 + k_2k_3}{k_{-3}(k_{-2} + k_2 + k_3)}$
v_{max}^f	$\frac{k_1k_2k_3}{k_{-1}k_{-2} + k_{-1}k_3 + k_2k_3} e_{tot}$
v_{max}^b	$\frac{k_{-1}k_{-2}k_{-3}}{k_{-1}k_{-2} + k_{-1}k_3 + k_2k_3} e_{tot}$

The same procedure can be carried out to derive equivalent Michaelis-Menten constants for the higher-order elementary reactions. King and Altman [36] and Wang and Hanes [37] also proposed two simple approaches to derive Michaelis-Menten representations in terms of the individual rate constants without explicitly solving the algebraic equations.

The optimal combination of the elementary kinetic parameters was identified for the mutants grown aerobically with glucose in the first step of the optimization problem. This

is equivalent of estimating K_m and v_{max} values. In the second step of the optimization problem, the identified elementary kinetic parameters were fixed and the total level of each enzyme e_0 was estimated under the remaining three mutant conditions, separately. This is equivalent of fixing K_m's to the estimated values at the first step while estimating v_{max} values under the other three conditions, independently.

Supplementary References

1. Schomburg I, Chang A, Placzek S, Sohngen C, Rother M, Lang M, Munaretto C, Ulas S, Stelzer M, Grote A *et al*: **BRENDA in 2013: integrated reactions, kinetic data, enzyme function data, improved disease classification: new options and contents in BRENDA**. *Nucleic acids research* 2013, **41**(Database issue):D764-772.
2. Keseler IM, Mackie A, Peralta-Gil M, Santos-Zavaleta A, Gama-Castro S, Bonavides-Martinez C, Fulcher C, Huerta AM, Kothari A, Krummenacker M *et al*: **EcoCyc: fusing model organism databases with systems biology**. *Nucleic acids research* 2013, **41**(Database issue):D605-612.
3. Leighty RW, Antoniewicz MR: **Parallel labeling experiments with [U-13C]glucose validate E. coli metabolic network model for 13C metabolic flux analysis**. *Metabolic engineering* 2012, **14**(5):533-541.
4. Cleland WW: **The kinetics of enzyme-catalyzed reactions with two or more substrates or products. I. Nomenclature and rate equations**. *Biochimica et biophysica acta* 1963, **67**:104-137.
5. Ishii N, Nakahigashi K, Baba T, Robert M, Soga T, Kanai A, Hirasawa T, Naba M, Hirai K, Hoque A *et al*: **Multiple high-throughput analyses monitor the response of E. coli to perturbations**. *Science* 2007, **316**(5824):593-597.
6. Khodayari A, Zomorrodi AR, Liao JC, Maranas CD: **A kinetic model of Escherichia coli core metabolism satisfying multiple sets of mutant flux data**. *Metabolic engineering* 2014, **25**:50-62.
7. Kabir MM, Ho PY, Shimizu K: **Effect of ldhA gene deletion on the metabolism of Escherichia coli based on gene expression, enzyme activities, intracellular metabolite concentrations, and metabolic flux distribution**. *Biochem Eng J* 2005, **26**(1):1-11.
8. Zhao J, Baba T, Mori H, Shimizu K: **Global metabolic response of Escherichia coli to gnd or zwf gene-knockout, based on 13C-labeling experiments and the measurement of enzyme activities**. *Applied microbiology and biotechnology* 2004, **64**(1):91-98.

9. Zhao J, Shimizu K: **Metabolic flux analysis of Escherichia coli K12 grown on 13C-labeled acetate and glucose using GC-MS and powerful flux calculation method.** *Journal of biotechnology* 2003, **101**(2):101-117.
10. Stelling J, Klamt S, Bettenbrock K, Schuster S, Gilles ED: **Metabolic network structure determines key aspects of functionality and regulation.** *Nature* 2002, **420**(6912):190-193.
11. Kabir MM, Shimizu K: **Metabolic regulation analysis of icd-gene knockout Escherichia coli based on 2D electrophoresis with MALDI-TOF mass spectrometry and enzyme activity measurements**. *Applied microbiology and biotechnology* 2004, **65**(1):84-96.
12. Peng L, Shimizu K: **Effect of fadR gene knockout on the metabolism of Escherichia coli based on analyses of protein expressions, enzyme activities and intracellular metabolite concentrations**. *Enzyme and microbial technology* 2006, **38**(3):512-520.
13. Yang C, Hua Q, Baba T, Mori H, Shimizu K: **Analysis of Escherichia coli anaplerotic metabolism and its regulation mechanisms from the metabolic responses to altered dilution rates and phosphoenolpyruvate carboxykinase knockout**. *Biotechnology and bioengineering* 2003, **84**(2):129-144.
14. Hoque MA, Siddiquee KAZ, Shimizu K: **Metabolic control analysis of gene-knockout Escherichia coli based on the inverse flux analysis with experimental verification**. *Biochemical engineering journal* 2004, **19**(1):53-59.
15. Kadir TA, Mannan AA, Kierzek AM, McFadden J, Shimizu K: **Modeling and simulation of the main metabolism in Escherichia coli and its several single-gene knockout mutants with experimental verification**. *Microbial cell factories* 2010, **9**:88.
16. Chassagnole C, Noisommit-Rizzi N, Schmid JW, Mauch K, Reuss M: **Dynamic modeling of the central carbon metabolism of Escherichia coli**. *Biotechnology and bioengineering* 2002, **79**(1):53-73.
17. Bennett BD, Kimball EH, Gao M, Osterhout R, Van Dien SJ, Rabinowitz JD: **Absolute metabolite concentrations and implied enzyme active site occupancy in Escherichia coli**. *Nature chemical biology* 2009, **5**(8):593-599.
18. Haverkorn van Rijsewijk BR, Nanchen A, Nallet S, Kleijn RJ, Sauer U: **Large-scale 13C-flux analysis reveals distinct transcriptional control of respiratory and fermentative metabolism in Escherichia coli**. *Molecular systems biology* 2011, **7**:477.
19. Fendt SM, Oliveira AP, Christen S, Picotti P, Dechant RC, Sauer U: **Unraveling condition-dependent networks of transcription factors that control metabolic pathway activity in yeast**. *Molecular systems biology* 2010, **6**:432.
20. Leyn SA, Li X, Zheng Q, Novichkov PS, Reed S, Romine MF, Fredrickson JK, Yang C, Osterman AL, Rodionov DA: **Control of proteobacterial central carbon metabolism by the HexR transcriptional regulator: a case study**

in Shewanella oneidensis. *The Journal of biological chemistry* 2011, **286**(41):35782-35794.

21. Tannler S, Fischer E, Le Coq D, Doan T, Jamet E, Sauer U, Aymerich S: **CcpN controls central carbon fluxes in Bacillus subtilis**. *Journal of bacteriology* 2008, **190**(18):6178-6187.
22. Kochanowski K, Sauer U, Chubukov V: **Somewhat in control--the role of transcription in regulating microbial metabolic fluxes**. *Current opinion in biotechnology* 2013, **24**(6):987-993.
23. Salmon KA, Hung SP, Steffen NR, Krupp R, Baldi P, Hatfield GW, Gunsalus RP: **Global gene expression profiling in Escherichia coli K12: effects of oxygen availability and ArcA**. *The Journal of biological chemistry* 2005, **280**(15):15084-15096.
24. Constantinidou C, Hobman JL, Griffiths L, Patel MD, Penn CW, Cole JA, Overton TW: **A reassessment of the FNR regulon and transcriptomic analysis of the effects of nitrate, nitrite, NarXL, and NarQP as Escherichia coli K12 adapts from aerobic to anaerobic growth**. *The Journal of biological chemistry* 2006, **281**(8):4802-4815.
25. Choudhary MK, Yoon JM, Gonzalez R, Shanks JV: **Re-examination of metabolic fluxes in Escherichia coli during anaerobic fermentation of glucose using C-13 labeling experiments and 2-dimensional nuclear magnetic resonance (NMR) spectroscopy**. *Biotechnol Bioproc E* 2011, **16**(3):419-437.
26. Salmon K, Hung SP, Mekjian K, Baldi P, Hatfield GW, Gunsalus RP: **Global gene expression profiling in Escherichia coli K12. The effects of oxygen availability and FNR**. *The Journal of biological chemistry* 2003, **278**(32):29837-29855.
27. Cox SJ, Shalel Levanon S, Bennett GN, San KY: **Genetically constrained metabolic flux analysis**. *Metabolic engineering* 2005, **7**(5-6):445-456.
28. Kang Y, Weber KD, Qiu Y, Kiley PJ, Blattner FR: **Genome-wide expression analysis indicates that FNR of Escherichia coli K-12 regulates a large number of genes of unknown function**. *Journal of bacteriology* 2005, **187**(3):1135-1160.
29. Sauer U, Canonaco F, Heri S, Perrenoud A, Fischer E: **The soluble and membrane-bound transhydrogenases UdhA and PntAB have divergent functions in NADPH metabolism of Escherichia coli**. *The Journal of biological chemistry* 2004, **279**(8):6613-6619.
30. Valentini G, Stoppini M, Speranza ML, Malcovati M, Ferri G: **Bacterial pyruvate kinases have a shorter N-terminal domain**. *Biological chemistry Hoppe-Seyler* 1991, **372**(2):91-93.
31. Campos-Bermudez VA, Bologna FP, Andreo CS, Drincovich MF: **Functional dissection of Escherichia coli phosphotransacetylase structural domains and analysis of key compounds involved in activity regulation**. *The FEBS journal* 2010, **277**(8):1957-1966.
32. Izui K, Taguchi M, Morikawa M, Katsuki H: **Regulation of Escherichia coli phosphoenolpyruvate carboxylase by multiple effectors in vivo. II.**

Kinetic studies with a reaction system containing physiological concentrations of ligands. *Journal of biochemistry* 1981, **90**(5):1321-1331.
33. Joshi VC, Wakil SJ: **Studies on the mechanism of fatty acid synthesis. XXVI. Purification and properties of malonyl-coenzyme A--acyl carrier protein transacylase of Escherichia coli**. *Archives of biochemistry and biophysics* 1971, **143**(2):493-505.
34. Brown G, Singer A, Lunin VV, Proudfoot M, Skarina T, Flick R, Kochinyan S, Sanishvili R, Joachimiak A, Edwards AM *et al*: **Structural and biochemical characterization of the type II fructose-1,6-bisphosphatase GlpX from Escherichia coli**. *The Journal of biological chemistry* 2009, **284**(6):3784-3792.
35. Ogawa T, Murakami K, Mori H, Ishii N, Tomita M, Yoshin M: **Role of phosphoenolpyruvate in the NADP-isocitrate dehydrogenase and isocitrate lyase reaction in Escherichia coli**. *Journal of bacteriology* 2007, **189**(3):1176-1178.
36. King ELaA, C.: **A Schematic Method of Deriving the Rate Laws for Enzyme-Catalyzed Reactions**. *J Phys Chem* 1956, **60**:4.
37. Cornish-Bowden A: **Fundamentals of Enzyme Kinetics**, 4 edn: Wiley; 2012.
